# Mutations in nuclear genes encoding mitochondrial ribosome proteins restore pollen fertility in S male-sterile maize

**DOI:** 10.1093/g3journal/jkae201

**Published:** 2024-08-20

**Authors:** Yan Wang, Rosalind Williams-Carrier, Robert Meeley, Timothy Fox, Karen Chamusco, Mina Nashed, L Curtis Hannah, Susan Gabay-Laughnan, Alice Barkan, Christine Chase

**Affiliations:** Horticultural Sciences Department, University of Florida, Gainesville, FL 32611, USA; Institute of Molecular Biology, University of Oregon, Eugene, OR 97403, USA; Corteva AgriScience (retired), Johnston, IA 50131, USA; Corteva AgriScience (retired), Johnston, IA 50131, USA; Horticultural Sciences Department, University of Florida, Gainesville, FL 32611, USA; Horticultural Sciences Department, University of Florida, Gainesville, FL 32611, USA; Horticultural Sciences Department, University of Florida, Gainesville, FL 32611, USA; Department of Plant Biology, University of Illinois, Urbana, IL 61801, USA; Institute of Molecular Biology, University of Oregon, Eugene, OR 97403, USA; Horticultural Sciences Department, University of Florida, Gainesville, FL 32611, USA

**Keywords:** cytoplasmic male sterility, fertility restoration, plant mitochondria, ribosome protein, *Zea mays*

## Abstract

The interaction of plant mitochondrial and nuclear genetic systems is exemplified by mitochondria-encoded cytoplasmic male sterility (CMS) under the control of nuclear *restorer-of-fertility* genes. The S type of CMS in maize is characterized by a pollen collapse phenotype and a unique paradigm for fertility restoration in which numerous nuclear *restorer-of-fertility lethal* mutations rescue pollen function but condition homozygous-lethal seed phenotypes. Two nonallelic restorer mutations recovered from *Mutator* transposon-active lines were investigated to determine the mechanisms of pollen fertility restoration and seed lethality. *Mu* Illumina sequencing of transposon-flanking regions identified insertion alleles of nuclear genes encoding mitochondrial ribosomal proteins RPL6 and RPL14 as candidate *restorer-of-fertility lethal* mutations. Both candidates were associated with lowered abundance of mitochondria-encoded proteins in developing maize pollen, and the *rpl14* mutant candidate was confirmed by independent insertion alleles. While the restored pollen functioned despite reduced accumulation of mitochondrial respiratory proteins, normal-cytoplasm plants heterozygous for the mutant alleles showed a significant pollen transmission bias in favor of the nonmutant *Rpl6* and *Rpl14* alleles. CMS-S fertility restoration affords a unique forward genetic approach to investigate the mitochondrial requirements for, and contributions to, pollen and seed development.

## Introduction

Flowering plants, like other eukaryotes, depend upon mitochondria for respiration and the production of ATP (Reviewed by Fernie *et al.* 2004; Braun 2020). Mitochondria are also an important source of biosynthetic intermediates (Geigenberger and Fernie 2014; Zhang and Fernie 2023) and act as sensing and signaling centers (Sweetlove *et al.* 2007; Jacoby *et al.* 2012; Schwarzländer and Finkemeier 2013; Van Aken 2021; Welchen *et al.* 2021). The biogenesis and function of these essential organelles depends on the combined and coordinated action of mitochondrial and nuclear genetic systems. Mitochondrial genomes encode only a subset of the respiratory protein complex subunits and of the apparatus needed for mitochondrial gene expression (Sugiyama *et al.* 2005; Kubo and Newton 2008). Genes encoding respiratory proteins and proteins required for the regulation and execution of mitochondrial transcription, RNA metabolism, translation, protein complex assembly, and turnover are divided between mitochondrial and nuclear genomes, with mitochondrial import of the nuclear-encoded proteins (Murcha *et al.* 2014; Møller *et al.* 2021; Ghifari *et al.* 2023).

The cytoplasmic male sterility (CMS) trait of flowering plants is a mitochondria-encoded failure to produce or release functional pollen. CMS is commonly conditioned by the gain of novel mitochondrial genes or, occasionally, mutant variants of mitochondrial respiratory genes (Hanson 1991; Linke and Börner 2005; Chase 2007; Carlsson *et al.* 2008; Horn *et al.* 2014). CMS can be reversed or suppressed by cytoplasm-specific, nuclear *restorer-of-fertility* (*restorer*) genes. The multiple, independently evolved CMS systems are characterized by a diversity of male sterility mechanisms, causal mitochondrial genes, and nuclear-encoded fertility restoration systems (Chase 2007; Chen and Liu 2014; Anisimova 2020; Kubo *et al.* 2020; Gautam *et al.* 2023; Kitazaki *et al.* 2023). Many restorer genes encode members of the large pentatricopeptide repeat (PPR) protein family (Chen and Liu 2014; Kubo *et al.* 2020). Most PPR proteins are targeted to plastids or mitochondria, where they function as site-specific RNA binding proteins that mediate key organelle gene expression processes including transcript stabilization, intron splicing, the C-to-U RNA editing process required for correct protein coding, and translation (Barkan and Small 2014). Restorer and restorer-like PPR proteins comprise a separate clade of PPRs consistent with adaptive, gain-of-function for the silencing of specific mitochondrial CMS genes (Fujii *et al.* 2011; Dahan and Mireau 2013). Other restorer genes encode an aldehyde dehydrogenase (Cui *et al.* 1996), a helix-loop-helix transcription factor (Jaqueth *et al.* 2020), a mitochondrial transcript termination factor (Hackauf *et al.* 2017; Bernhard *et al.* 2019), a metalloprotease (Matsuhira *et al.* 2012), and a mitochondrial glycine-rich protein (Itabashi *et al.* 2011). Glycine-rich proteins interact with PPR proteins in fertility restoration (Hu *et al.* 2012, 2013). In addition, a reduced-expression allele of the *RETROGRADE-REGULATED MALE STERILITY* (*RMS*) locus restores fertility to pollen of the CW-type CMS in rice. RMS encodes a mitochondria-targeted, 178-amino acid protein with possible acyl carrier protein synthase function. RMS conditions pollen sterility when highly expressed in the CW CMS background (Fujii and Toriyama 2009; Suketomo *et al.* 2020). Collectively, CMS fertility restoration systems provide insights into nuclear-mitochondrial cross talk in plants and potential routes to modify organelle genome function and plant phenotype.

In CMS type S (CMS-S) of *Zea mays* L. (maize), pollen collapse is conditioned by expression of a mitochondrial open reading frame *orf355* and compromised activity of respiratory complex I (Xiao *et al.* 2020, 2023). CMS-S pollen carrying a restorer allele will develop normally, whereas pollen carrying a nonrestoring allele will collapse (Buchert 1961). The restorers *Rf3* (Buchert 1961) and *Rf9* (Gabay-Laughnan *et al.* 2009) are common within maize germplasm. *Rf3* encodes a PPR protein associated with decreased RNA editing, increased internal cleavage and decreased abundance of *orf355* transcripts (Qin *et al.* 2021). Additional CMS-S restorers, however, arise through genetic mutations (Laughnan and Gabay 1973; Gabay-Laughnan *et al.* 2018). While these restorer mutations rescue CMS-S pollen function, many condition a homozygous-lethal phenotype with respect to seed development. These *restorer-of-fertility lethal* (*rfl*) alleles result from loss-of-function mutations that are hypothesized to disrupt expression of CMS-S in the pollen at the expense of broader mitochondrial functions that are essential for seed, but not pollen, development (Wen *et al.* 2003). A collection of *rfl* mutants was recovered from CMS-S *Mutator* (*Mu*) transposon-active maize stocks to facilitate the molecular cloning and identification of *rfl* genetic loci (Gabay-Laughnan *et al.* 2018). Here, Illumina sequencing of *Mu* transposon-flanking regions (*Mu* Illumina) (Williams-Carrier *et al.* 2010) identified two nonallelic *rfl* mutations as *Mu* transposon insertions in nuclear genes encoding the mitochondrial ribosomal proteins RPL6 and RPL14.

## Methods

### Genetic nomenclature

In maize genetic nomenclature (http://maizegdb.org/maize_nomenclature.php, accessed 2023 09 05), loci and recessive alleles at a locus are designated by a lowercase italic gene symbol. The first letter of the gene symbol is capitalized to indicate a dominant allele at the locus. In the case of gene families, the gene symbol also includes a number or lowercase letter to distinguish among family members. Gene symbols are followed by *-allele designator* for mutant alleles or *-stock of origin* for nonmutant alleles. Corresponding proteins are indicated in nonitalicized, capital letters. Transposon insertion alleles are designated by *gene symbol::transposon-allele designator*. Unknown loci of maize are indicated by the symbol*. The *rfl* mutants at unknown loci are named rfl*-allele designator and corresponding nonmutant, nonrestoring alleles are designated Rfl*-allele designator. In describing genetic crosses, the maternal (seed) parent is written first.

### Genetic materials

The maize stocks used in this study are summarized in Supplementary Table 1 of Supplementary File 1. Plants were field grown in Citra, FL or Champaign, IL, or grown in greenhouse pot culture under ambient light in Gainesville, FL. The two independent, nonallelic *rfl* mutants investigated in this study, rfl*-04-229 and rfl*-04-230, were recovered from a screen of CMS-S plants crossed with a nonrestoring, color-converted B73 (ccB73) *Mu*-on stock (Gabay-Laughnan *et al.* 2018). Crosses were made with rfl*-04-229/Rfl*-04-229 or with rfl*-04-230/Rfl*-04-230 plants (Supplementary Fig. 1 in Supplementary File 2) to produce two pairs of first cousins for each mutant. DNA extracted from these plants was analyzed by *Mu* Illumina as described by Williams-Carrier *et al.* (2010) to identify *Mu* insertions that were present in all four mutant relatives, thereby defining candidate rfl*-04-229 and rfl*-04-230 mutant loci. Additional alleles were sought from the Photosynthetic Mutant Library (Belcher *et al.* 2015), W22 UniformMu (McCarty *et al.* 2013) and TUSC (Meeley and Briggs 1995) reverse genetics resources and recovered in the case of rfl*-04-230.

### Plant genotyping

Total genomic DNA was extracted from leaf tissue as described by Grosser *et al.* (2023). The oligonucleotide primers used for PCR amplification of maize genomic DNA are listed in Supplementary Table 2 of Supplementary File 1. *Mu*-flanking sequences were amplified between the *Mu* terminal inverted repeat (TIR) primer and gene-specific primers. The corresponding regions of noninsertion alleles were amplified with the gene-specific primer pairs. PCR was performed in a 25 µL reaction mixture containing 1X GoTaq Hot-Start master mix (Promega Corp.), 20 ng of DNA template, and 0.2 µM each of the forward and reverse primers. Direct PCR of seedling leaf samples was used for transmission bias studies. Tissue pieces were punched with a 1000 µl pipet tip into tubes containing 60 µl of 2X Terra PCR Direct buffer (Clontech Laboratories, Inc.). Samples were snap frozen at −80°C and thawed at 98°C. Tissue was pelleted for 10 min in a microcentrifuge and 2 µl of each recovered supernatant was used as PCR template. PCR reactions of 30 ul contained Terra Direct hot-start master mix, 0.6 units of Terra PCR Direct Polymerase and 0.2 µM each of the forward and reverse primers. All genotyping PCR amplifications were initiated with a 2 min 98°C denaturation step followed by 33 cycles of 98°C for 10 s, 62°C for 15 s, and 72°C for 90 s, with a final extension at 72°C for 10 min. PCR products were fractionated by electrophoresis through 2% (w/v) agarose gels, stained and imaged as described previously (Grosser *et al.* 2023). Genetic mapping with maize bin 3.04 markers was carried out with *umc2000*, *umc2158*, *umc139*2, *umc0132*, *umc2262,* and *umc2263* microsatellite primer sets. These primer sequences are available from the Maize Genome Database (Harper *et al.* 2016) (http://www.maizegdb.org, accessed 2023 09 05). Microsatellite markers were PCR-amplified, fractionated by electrophoresis through 5% or 10% Criterion TBE precast polyacrylamide gels (Bio-Rad Laboratories, Inc.) and imaged as described by López *et al.* (2014).

### DNA sequencing

PCR-amplified genomic DNA regions were sequenced directly. PCR primers were removed by centrifugation through 30 or 50 kDa spin filtration devices. Sanger sequencing was performed by the University of Florida Interdisciplinary Center for Biotechnology Research (ICBR) DNA sequencing core laboratory. The CLUSTAL W program (Thompson *et al*. 1994), accessed through the San Diego Biology Workbench (Subramaniam 1998, http://workbench.sdsc.edu, accessed 2015 03 04) was used to align sequences for confirmation of *Mu* insertion sites and identification of single nucleotide polymorphisms (SNPs) in *Mu* TIRs and insertion-flanking regions.

### cDNA amplification and sequencing

**Fig. 1.**
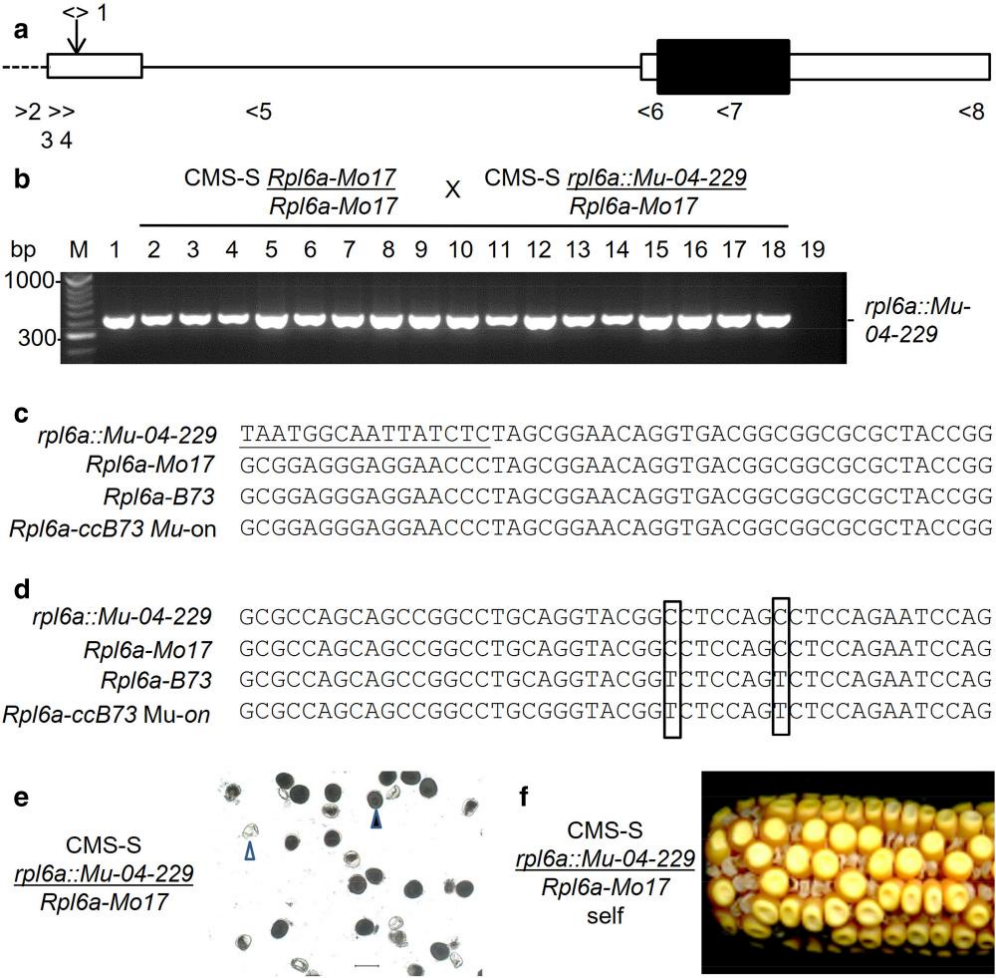
The rfl*-04-229 candidate allele *rpl6a::Mu-04-229* is linked to fertility restoration and contains a *Mu* insert in the *Rpl6a-Mo17* 5′ UTR. a) The *rpl6a* (GRMZM2G080608-Zm00001d051422) gene model (http://www.gramene.org/, release 42, accessed 2014 12 02). This gene model was absent in subsequent releases. Boxes represent exons and filled boxes coding sequences. Solid lines represent introns and broken lines gene flanking sequences. The downward arrow marks the *Mu* insertion point. Right and left arrows with numbers indicate forward and reverse primers, respectively. b) PCR genotyping for a *Mu* TIR-*rpl6a* amplicon in a CMS-S *Rpl6a-Mo17/Rpl6a-Mo17* X CMS-S *rpl6a::Mu-04-229/Rpl6a-Mo17* progeny, all expected to inherit the paternal restoring allele. PCR primers were 1 and 5 in (a), above. M, 1 and 19 designate marker, pollen parent, and negative PCR control lanes, respectively. Lanes 2-18 show amplification products from 17 random progeny all carrying the paternal *rpl6a::Mu-04-229* allele. (c, d) *Rpl6a* 5′ UTRs amplified from total genomic DNAs with primers 1 and 5 (*rpl6a::Mu-04-229*) or 2 and 5 (*Rpl6a-Mo17, Rpl6a-B73* and *Rpl6a::ccB73 Mu*-on), sequenced directly and aligned. Two segments of the alignment show a *Mu* terminus (underlined) adjacent to *Rpl6a* sequences (c) and two SNPs (boxed) that distinguished *Rpl6a-Mo17* and *rpl6a::Mu-04-229* alleles from *Rpl6-B73* (d). For the full sequence alignment see Supplementary Fig. 2 in Supplementary File 2. e) A pollen sample produced by a CMS-S *rpl6a::Mu-04-229/Rpl6a-Mo17* plant from the family analyzed in (b). Open arrow heads designate collapsed pollen and filled arrows restored pollen. The scale bar represents 100 µm. f) Segregating mutant and normal kernels produced by self-pollination of a CMS-S *rpl6a::Mu-04-229/Rpl6a-Mo17* plant from the family analyzed in (b).

**Fig. 2.**
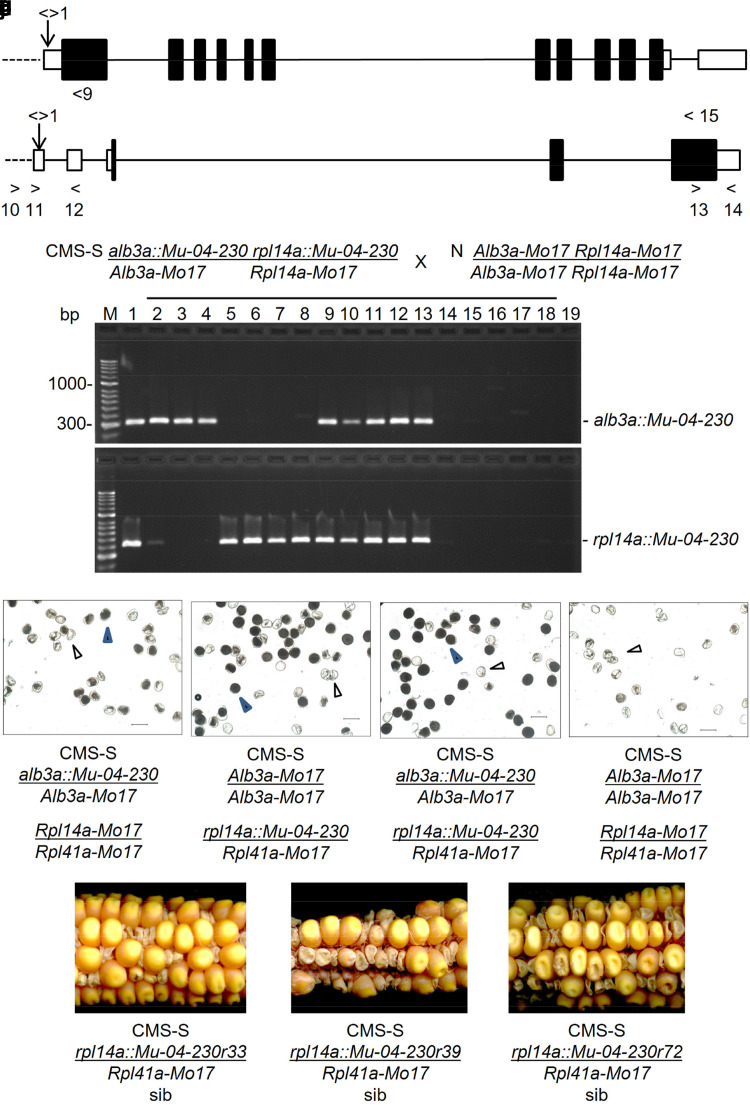
Genetic recombination separates the linked *alb3::Mu* and *rpl14a::Mu* insertions and identifies *rpl14a::Mu-04-230* as a restorer gene candidate (a) the *alb3a* (GRMZM5G839422-Zm00001d039852) gene model (http://ensembl.gramene.org/Zea_mays, accessed 2017 7 14). Boxes represent exons and filled boxes coding sequences. Solid lines represent introns and broken lines gene flanking sequences. The downward arrow marks the *Mu* insertion. Right and left arrows with numbers indicate forward and reverse primers, respectively. b) The *rpl14a* (GRMZM2G098957-Zm00001d041322) gene model accessed and labeled as described for (a), above. c) PCR genotyping for the *alb3a::Mu-04-230* and *rpl14a::Mu-04-230* insertion alleles in a CMS-S *alb3a::Mu-04-230_rpl14a::Mu-04-230/Alb3a-Mo17_Rpl14a-Mo17* × N-cytoplasm *Alb3a-Mo17_Rpl14a-Mo17* progeny. Genotyping for the *alb3a::Mu-04-230* allele with primers 1 and 9 (a, above) is shown in the top panel. Genotyping the same progeny for the *rpl14a::Mu-04-230* allele with primers 1 and 12 (b, above) is shown in the lower panel. M, 1 and 19 designate marker, positive, and negative PCR control lanes, respectively. Lanes 2–4 and 5–9 show PCR products from recombinant progeny carrying only the *alb3a::Mu-04-230* insertion or only the *rpl14a::Mu-04-230* insertion, respectively. Lanes 9–13 and 14–18 show PCR products from plants carrying both insertions or neither insertion, respectively. (d–g) Pollen samples from CMS-S plants carrying the *alb3a::Mu-04-230* insertion allele, the *rpl14a::Mu-04-230* insertion allele, both insertions or neither insertion, respectively. Open arrows designate collapsed pollen and filled arrows normal pollen. The scale bar represents 100 µm. (h–j) Progeny ears from sib pollinations within stocks established from three recombinants carrying only the *rpl14a::Mu-04-230* allele (lanes 6–8 in c, above) each segregating for a seed-lethal phenotype.

Gene transcripts were investigated by amplification and sequencing of cDNAs synthesized on total RNA samples extracted from pollen. The starch-filled, restored pollen was physically separated from the collapsed, nonrestored pollen by pelleting through 70% sucrose as described by Gabay-Laughnan *et al.* (2018). Pollen recovered from normal (N) cytoplasm tassels was also pelleted through 70% sucrose to recover the comparable developmental stage of control pollen for molecular comparisons. Pollen pellets of 0.25 g were frozen and stored at −80°C until they were used for extraction of total RNA as described by Wen and Chase (1999). The SuperScript First-Strand Synthesis System (Thermo Fisher Scientific) was used to create oligo(d)-primed first-strand cDNAs. The *rpl6a* and *rpl14a* cDNAs were then amplified in 50 µl reactions containing, 1.5 units of TaKaRa Ex Taq Hot-Start DNA Polymerase (Takara Bio USA, Inc.), 1X TaKaRa Ex Taq Hot-Start buffer, 2 mM MgCl_2_, dNTPs at 0.2 mM each, 1 µl of first-strand DNA synthesis reaction and gene-specific forward and reverse primers at 0.2 µM of each. Primer positions are shown in Figs. 1 and 2, and primer sequences are listed in Supplementary Table 2 of Supplementary File 1. Amplification was for 30 cycles of 94°C for 1 min, 55°C for 2 min, and 73°C for 3 min. PCR primers were removed as described for genomic DNA amplifications above. PCR products were sequenced by Eurofins Genomics US and sequence alignments were performed on the MultAlin server (Corpet 1988) (http://multalin.toulouse.inra.fr/multalin/, accessed 2022 09 02). The protein coding of cDNA sequences was analyzed with the EXPASY Translate tool (https://web.expasy.org/translate/, accessed 2023 09 05).

### Pollen phenotyping

Pollen fertility phenotypes of individual plants were determined by counting restored (starch-filled) and nonrestored (collapsed) pollen. Florets were harvested from the main rachis of fully emerged tassels and stored in 70% v/v ethanol. Anthers were dissected from the florets and crushed in 70% ethanol on a microscope slide. The released pollen was viewed and photographed with an LCD digital microscope at 40X magnification. The pollen phenotypes were determined by counting the collapsed and starch-filled pollen grains in the images. The percentage of restored pollen was determined from counts of at least 400 pollen grains per plant. CMS-S plants heterozygous for a restoring and a nonrestoring allele at a single locus are expected to produce 50% starch-filled and 50% collapsed pollen (Buchert 1961).

For protein phenotyping, tassels were collected from CMS-S plants heterozygous for restoring and nonrestoring alleles. The starch-filled, restored pollen and N-cytoplasm control pollen samples were recovered by pelleting through 70% sucrose as described above. Total detergent-soluble proteins were extracted from frozen pollen pellets, fractionated by denaturing gel electrophoresis, transferred to nitrocellulose, decorated with antibodies, and imaged as described previously (Chamusco *et al.* 2022). Antibodies, also described by Chamusco *et al.* (2022), were selected to give well-separated signals on blots of total pollen protein and to query different mitochondrial protein complexes. These were complex I, NADH dehydrogenase subunit 7 (NAD7); complex IV, cytochrome oxidase subunit 2 (COXII); and complex V, ATP synthase subunits 2, 6, and 8 (ATP2, ATP6, and ATP8), along with the alternative oxidase (AOX). Additionally, a monoclonal antibody recognizing maize mitochondrial HSP70 (Lund *et al.* 1998) was selected for quantitative reference because it is a nuclear-encoded, mitochondrial protein that is not a component of complex I, IV, V, or the AOX, so its abundance is not likely to be directly influenced by that of the other proteins studied. Images were captured and band volumes were determined with Image Lab software, version 6.1 (Bio-Rad Laboratories). Three biological replicates were analyzed, and statistical analyses were performed in Microsoft Excel (Microsoft Inc.).

### Seed phenotyping

Cytological analysis of mutant and normal maize kernels was performed 16 days after pollination (DAP). CMS-S *rpl6a-04-229*/*Rpl6a-Mo17* and CMS-S *rpl14a-04-230*/*Rpl14a-Mo17* plants were self or sib pollinated. By 16 DAP, homozygous-mutant kernels were readily distinguished by size from nonmutant kernels and from unpollinated ovules. Mutant and nonmutant sibling kernels excised from the same ear were cut bilaterally, fixed in 4% formaldehyde and 0.25% glutaraldehyde in phosphate buffered saline, dehydrated through an ethanol series and embedded in Paraplast Plus paraffin (Electron Microscopy Sciences). Tissue sections of 7 µm were adhered to poly-L-lysine coated slides and stained with toluidine blue O (TBO) stain (1% w/v TBO, 1% w/v Na_2_B_4_O_7_, dissolved in water and filtered before use). Sections were covered with TBO stain and heated at 60°C until the edges of the solution appeared metallic. The sections were rinsed with deionized water and dehydrated through a graded ethanol series and affixed to slides in SecureMount (Thermo Fisher Scientific Inc.). Whole kernel sections were viewed through a Leica MZ 12.5 microscope. Micrographs were taken with a CCD digital camera model 2.3.1 (Diagnostic Instruments Inc.) and captured using SPOT software v 5.1 (Spot Imaging Solutions, Diagnostic Instruments Inc.).

## Results

### Identification of *rfl* candidate genes

In the case of the CMS-S restorer mutant rfl*-04-229, *Mu* Illumina identified a single candidate insertion common to the four mutant individuals analyzed. This insertion was located in the 5′ untranslated region (UTR) of GRMZM2G080608/Zm00001d051422 on chromosome 4 (Fig. 1a). This gene model (http://www.gramene.org/, release 42, accessed 2014 12 02 but absent in subsequent releases) predicted a single transcript encoding a 104-amino acid RPL6. This protein was not strongly predicted to locate to the mitochondria by iPSORT (http://ipsort.hgc.jp/#predict, accessed 2014 12 02) (Bannai *et al.* 2002), or TargetP (https://services.healthtech.dtu.dk/services/TargetP-2.0/, accessed 2023 09 06) (Almagro Armenteros *et al.* 2019) (Supplementary Table 3 in Supplementary File 1). Nevertheless, BLASTp against nonplant proteins (https://blast.ncbi.nlm.nih.gov/Blast.cgi, accessed 2023 07 09) revealed the highest similarity to alpha proteobacterial L6 ribosomal proteins. Two paralogs, GRMZM2G86788/Zm00001d029201 located on chromosome 1 and GRMZM2G170870/Zm00001d047462 on chromosome 9, predicted a plastid-targeted RPL6, although transcript variant 2 of GRMZM2G86788 potentially encodes a mitochondria or cytosolic localized protein (Supplementary Table 3 in Supplementary File 1). We designated the GRMZM2G080608/Zm00001d051422 candidate locus as *rpl6a* and the candidate rfl*-04-229 restoring allele as *rpl6a::Mu-04-229*, and propose paralogs GRMZM2G86788 and GRMZM2G170870 be designated *rpl6b* and *rpl6c*, respectively. A 419 bp PCR amplicon generated between *Mu* TIR and *rpl6a* primers was genetically linked to the rfl*-04-229 mutation. In CMS-S rfl*-04-229/Rfl*04-229 plants, only pollen carrying the restoring (rfl*-04-229 mutant) allele will function. In two generations of crossing CMS-S *Rpl6a-Mo17*/*Rpl6a-Mo17* seed parents with CMS-S *rpl6a::Mu-04-229/Rpl6a-Mo17* pollen parents, 18 of 18 and 23 of 23 progeny carried the insertion allele (Fig. 1b). DNA sequencing confirmed the *Mu* TIR adjacent to *rpl6a* sequences in this amplicon (Fig. 1c). PCR amplification followed by sequencing of the *Mu* insertion region in the nonmutant *Rpl6a-Mo17*, *Rpl6a-B73,* and *Rpl6a-ccB73 Mu*-on alleles identified three SNPs that distinguished the Mo17 allele from the B73 or ccB73 *Mu*-on allele. All three Mo17 SNPs were present in the *Mu* TIR-*rpl6a* amplicon, demonstrating that *Mu* inserted into the *Rpl6a-Mo17* allele rather than the *Rpl6a-ccB73 Mu*-on allele in creating this candidate *rfl* mutation (Fig. 1d, Supplementary Fig. 2 in Supplementary File 2).

Pollen and seed phenotypes were investigated for CMS-S Mo17 × CMS-S *rpl6a::Mu-04-229/Rpl6a-Mo17* plants and their progeny. Ten CMS-S Mo17 × CMS-S *rpl6a::Mu-04-229/Rpl6a-Mo17* plants grown to maturity produced an average of 51.6 ± 8.8% starch-filled pollen (Fig. 1e, Table 1). Self-pollination or sib-pollination of CMS-S *rpl6a::Mu-04-229/Rpl6a-Mo17* plants produced ears segregating an average of 44.2 ± 5.2% aborted seeds, as expected when both mutant and nonmutant eggs function, only the pollen carrying the restorer mutation functions, and the restorer mutation conditions a homozygous-lethal seed phenotype (Table 2).

**Table 1.** Pollen phenotypes of n-cytoplasm, CMS-S, and restored CMS-S plants.

Cytoplasm	Nuclear genotype	Pollen phenotype	
		*n^a^*	% normal*^b^*
CMS-S	*Rpl6a-Mo17/Rpl6a-Mo17; Rpl14a-Mo17/Rpl14a-Mo17*	4	5.6 ± 4.3
N*^c^*	*Rpl6a-Mo17/Rpl6a-Mo17; Rpl14a-Mo17/Rpl14a-Mo17*	4	93.9 ± 1.4
CMS-S	*rpl6a::Mu04-229/Rpl6a-Mo17*; *Rpl14a-Mo17/Rpl14a-Mo17*	8	51.6 ± 8.8
CMS-S	*Rpl6a-Mo17/Rpl6a-Mo17; rpl14a::Mu-04-229r33/Rpl14a-Mo17*	4	50.6 ± 5.1
CMS-S	*Rpl6a-Mo17/Rpl6a-Mo17; rpl14a::Mu-04-229r39/Rpl14a-Mo17*	4	46.9 ± 3.3
CMS-S	*Rpl6a-Mo17/Rpl6a-Mo17; rpl14a::Mu-04-229r72/Rpl14a-Mo17*	3	58.3 ± 9.6
CMS-S	*Rpl6a-Mo17/Rpl6a-Mo17; rpl14a::Mu-1021756/Rpl14a-Mo17*	10	52.9 ± 8.6
CMS-S	*Rpl6a-Mo17/Rpl6a-Mo17; rpl14a::Mu-PV03 41 D-05/Rpl14a-Mo17*	11	40.6 ± 16.1

^*a*^*n*, the number of plants that were phenotyped.

^*b*^The mean % of normal pollen (±SD) per plant, was determined by counting the number of normal (starch-filled) and aborted (empty, collapsed) pollen grains in samples of at least 400 grains taken from individual plants; means and standard deviations were calculated in Microsoft Excel (Microsoft, Inc.).

^*c*^N, normal-cytoplasm.

**Table 2.** Seed phenotypes on CMS-S ears segregating for *rfl* alleles.

Seed parent genotype	Pollen genotype	Progeny seed phenotype	
		n*^a^*	% mutant*^b^*
*rpl6a::Mu-04-229/Rpl6a-Mo17*	*rpl6a::Mu-04-229*	7	44.2 ± 5.2
*rpl14a::Mu-04-230r33/Rpl14a-Mo17*	*rpl14a::Mu-04-230r33*	7	50.3 ± 2.7
*rpl14a::Mu-04-230r39/Rpl14a-Mo17*	*rpl14a::Mu-04-230r39*	6	51.2 ± 4.6
*rpl14a::Mu-04-230r72/Rpl14a-Mo17*	*rpl14a::Mu-04-230r72*	3	48.3 ± 7.6
*rpl14a::Mu-1021756/Rpl14a-Mo17*	*rpl14a::Mu-1021756*	3	49.1 ± 3.9
*rpl14a::Mu-04-230r33/Rpl14a-Mo17*	*rpl14a::Mu-1021756*	3	46.4 ± 8.8
*rpl14a::mu-PV03 41 D-05/Rpl14a-Mo17*	*rpl14a::Mu-PV03 41 D-05*	6	37.5 ± 5.2
*rpl14a::mu-PV03 41 D-05/Rpl14a-Mo17*	*rpl14a::Mu-04-230r39*	3	38.3 ± 2.9
*rpl14a::mu-PV03 41 D-05/Rpl14a-Mo17*	*rpl14a::Mu-1021756*	2	47.2 ± 0.1 (46.8–47.6)

^*a*^*n*, the total number of progeny ears analyzed.

^*b*^The mean % of aborted seeds (±SD) per ear, was determined by counting all normal (starch-filled) and mutant (empty, collapsed, or small) seeds on each ear. Standard deviations were calculated in Microsoft Excel (Microsoft Inc.) The mean (range) is reported for *n* < 3.

In the case of the rfl*-04-230 mutation, *Mu* Illumina identified no candidate insertions present in all four mutant plants analyzed, but two insertions were present in three out of the four mutant plants. These candidates were pursued due to the possibility that a different restorer mutation created the plant that lacked the candidate insertions. Both candidate insertions, GRMZM5G839422/Zm00001d039852 (Fig. 2a) and GRMZM2G098957/Zm00001d041322 (Fig. 2b), were located on the short arm of chromosome 3. GRMZM5G839422 encodes a member of the OXA1/ALB3/YidC membrane protein insertion chaperone family. Targeting prediction programs variably predicted a mitochondrial or plastid location for this candidate gene product (Supplementary Table 4 in Supplementary File 1), while a BLASTp analysis (http://blast.ncbi.nlm.nih.gov/Blast.cgi, accessed 2015 01 11) demonstrated greatest similarity to the ALB3 thylakoid membrane chaperone family members. This locus was therefore designated *alb3a*, and the candidate rfl*-04-230 allele was designated *alb3a::Mu-04-230*. The second rfl*-04-230 candidate, GRMZM2G098957, predicted a ribosomal large subunit protein 14 (RPL14) with a mitochondrial targeting sequence (Supplementary Table 5 in Supplementary File 1). This locus was designated *rpl14a* and the candidate rfl*-04-230 restoring allele was designated *rpl14a::Mu-04-230*. The single paralog of *Rpl14a* (GRMZM5G804776) is located in the maize plastid genome (Supplementary Table 5 in Supplementary File 1).

The presence of two chromosome 3 insertions in the same rfl*-04-230 plants indicated that these insertions were likely positioned in cis and needed to be separated by recombination so that their contributions to fertility restoration could be assessed independently. A segregating family was generated for this purpose. A CMS-S plant heterozygous for both insertions (*alb3a::Mu-04-230_ rpl14a::Mu-04-230/Alb3a-Mo17_ Rpl14a-Mo17*) was fertilized with N-cytoplasm Mo17 pollen (*Alb3a-Mo17_ Rpl14a-Mo17*). Of 72 progeny, 69 were successfully examined for *Mu* insertion genotypes at both loci (Fig. 2c). Of these 69, 36 progeny carried neither of the insertion alleles and 26 progeny remained heterozygous for both insertions. The progeny also included three *alb3a::Mu-04-230_Rpl14a-Mo17* recombinants and four *Alb3a-Mo17_rpl14a::Mu-04-230* recombinants. Considering the recombinant plants and the 26 plants heterozygous for both insertions, the progeny segregated 1:1 for the presence or absence of each insertion. The Yates-corrected *X*^2^ values were 1.45 (*P* = 0.23) and 0.93 (*P* = 0.34) for the *alb3a-04-230* and *rpl14a Mu-04-230* insertions, respectively. Based upon the recovery of seven recombinants, the estimated recombination frequency for the 69.6 megabase pair (Mbp) interval between the two candidate loci was a relatively low 0.14 centiMorgans (cM) per Mbp.

Pollen phenotypes of the recombinant plants identified the GRMZM2G098957 *rpl14a::Mu-04-230* insertion as the stronger restorer candidate (Fig. 2d–g). Starch-filled and collapsed pollen counts were made for the seven recombinant plants, five representative homozygous noninsertion plants, and five representative plants heterozygous for insertions at both candidate loci (Table 3, Supplementary Table 6 in Supplementary File 1). As expected, the homozygous noninsertion plants produced almost no starch-filling pollen (an average of 1.5 ± 1.5%) and shed no pollen. The three *alb3a::Mu-04-230_Rpl14a-Mo17* recombinants produced indehiscent anthers that contained on average, 19.7 ± 3.9% starch-filled pollen. Of the four *Alb3a_rpl14a::Mu-04-230* recombinants, one off-type (late developing and stunted) plant produced 11% starch-filled pollen and was not propagated for further studies. The remaining three plants in this recombinant category (numbers 33, 39, and 72) produced, on average, 49.2 ± 4.0% starch-filled pollen fitting the expected segregation for restoring and nonrestoring alleles at a single locus. The five representative double heterozygote plants produced 63.4 ± 4.0% starch-filled pollen, indicating possible additional fertility restorers or modifiers that were not pursued further in this investigation.

**Table 3.** Recombination of *rpl14a::Mu* and *alb3a::Mu rfl* candidate loci.

Plant ID	Genotype*^a^*								% Starch- Filled Pollen*^b^*
	*alb3a*	*umc2000*	*umc2158*	*umc1392*	*umc0132*	*umc2262*	*umc2263*	*rpl14a*	
18	*::Mu/+^c^*	M/B*^d^*	M/M*^e^*	M/M	M/M	M/M	MM	*+/+^f^*	17
31	*::Mu/+*	M/B	M/B	M/M	M/M	M/M	M/M	*+/+*	18
63	*::Mu/+*	M/B	M/B	M/B	M/B	M/B	M/M	*+/+*	24
33	+/+	M/M	M/M	M/B	M/B	M/B	M/B	*::Mu*/+	50
08	+/+	M/M	M/M	M/M	M/B	M/B	M/B	*::Mu*/+	11
72	+/+	M/M	M/M	M/M	M/B	M/B	M/B	*::Mu*/+	50
39	+/+	M/M	M/M	M/M	M/M	M/M	M/M	*::Mu*/+	46
01	*::Mu/+*	M/B	M/B	M/B	M/B	M/B	M/B	*::Mu*/+	69
09	*::Mu/+*	M/B	M/B	M/B	M/B	M/B	M/B	*::Mu*/+	64
20	*::Mu/+*	M/B	M/B	M/B	M/B	M/B	M/B	*::Mu*/+	60
29	*::Mu/+*	M/B	M/B	M/B	M/B	M/B	M/B	*::Mu*/+	59
38	*::Mu/+*	M/B	M/B	M/B	M/B	M/B	M/B	*::Mu*/+	66
22	*+/+*	M/M	M/M	M/M	M/M	M/M	M/M	*+/+*	3
28	*+/+*	M/M	M/M	M/M	M/M	M/M	M/M	*+/+*	3
36	*+/+*	M/M	M/M	M/M	M/M	M/M	M/M	*+/+*	1
44	*+/+*	M/M	M/M	M/M	M/M	M/M	M/M	*+/+*	1
45	*+/+*	M/M	M/M	M/M	M/M	M/M	M/M	*+/+*	0

^*a*^A segregating population was created by fertilizing a CMS-S *alb3a::Mu-04-230 rpl14a::Mu-04-230/Alb3a-Mo17 Rpl14a-Mo17* plant with *Alb3a-Mo17 Rpl14a-Mo17* pollen, and progeny were genotyped for both *Mu* insertions. All recombinant plants (carrying only one of the two insertions), five representative nonmutant and five representative double-mutant segregants were genotyped for six microsatellite loci within the recombination interval.

^*b*^Determined by counting a minimum of 400 ethanol-fixed pollen grains per plant; pollen counts reported in Supplementary Table 6 of Supplementary File 1.

^*c*^*::Mu/+*, heterozygous for insertion and noninsertion (nonmutant) alleles.

^*d*^M/B, heterozygous for Mo17 and B73 alleles.

^*e*^M/M, homozygous for the Mo17 allele.

^*f*^+/+ homozygous for the noninsertion (nonmutant) allele.

The seven recombinant plants, five homozygous noninsertion plants, and five plants heterozygous for insertions at both candidate loci were also genotyped for six microsatellite markers that spanned the interval between the candidate loci (Table 3). This analysis demonstrated that the *alb3a::Mu-04-230* and *rpl14a::Mu-04-230* insertion alleles were linked to B73 microsatellite alleles. Therefore, both of these insertions likely occurred in the ccB73 *Mu*-on parent. This was confirmed in the case of *rpl14a::Mu-04-230*. DNA sequences immediately upstream of the *Mu* insertion site contained three SNPs that distinguished the Mo17 and ccB73 *Mu*-on parents, with the *rpl14a::Mu-04-230* sequence matching that of ccB73 (Supplementary Fig. 3 in Supplementary File 2). The microsatellite marker analysis defined recombination intervals for each of the seven recombination events. The recombination site was different for each of the *Alb3a-Mo17_rpl14a::Mu-04-230* recombinant plants. In the case of plant 39, the recombination break point was located between the *rpl14a::Mu-04-230* insertion and the closest marker tested, *umc2263*. Recombinant plants 33, 39, and 72 were each successfully crossed onto a CMS-S Mo17 seed parent, establishing three separate stocks for further studies. The mutant alleles carried by these stocks were designated *rpl14a::Mu-04-230r33, rpl14a::Mu-04-230r39, and rpl14a::Mu-04-230r72.*

Plants resulting from pollination of CMS-S Mo17 with each of the CMS-S *rpl14a::Mu-04-230* recombinant stocks had pollen and seed phenotypes consistent with an *rfl* allele. CMS-S plants heterozygous for the *rpl14a::Mu-04-230r33, rpl14a::Mu-04-230r39,* or *rpl14a::Mu-04-230r72* allele produced 50.6 ± 5.1, 46.9 ± 3.3 or 58.3 ± 9.6% starch-filled pollen, respectively (Table 1). Ears resulting from sib or self-pollinations of these heterozygotes segregated 50.3 ± 2.7, 51.2 ± 4.6 or 48.3 ± 7.6% seed-lethal phenotypes, respectively (Fig. 2h–j, Table 2).

### Confirmation of *rfl* candidate genes

In an attempt to confirm the *rpl6a::Mu-04-229* insertion allele as a restorer mutation, two publicly available, independent insertion alleles of GRMZM2G080608 were tested for fertility restoration activity. No insertions could be identified from the *Mu-*Illumina resource. A UniformMu (McCarty *et al.* 2013) insertion (mu1047993) located 56 bp 5′ of the *rpl6a::Mu-04-229* insertion, but still in the *rpl6a* 5′ UTR, did not create a seed-lethal phenotype or condition fertility restoration when crossed to CMS-S Mo17 plants. Insertions at this position in a gene often do not disrupt gene function. Thus, although genetic and phenotypic features of the *rpl6a::Mu-04-229* insertion were entirely consistent with its identification as an *rfl* allele, this candidate could not be confirmed with an independent mutant.

**Fig. 3.**
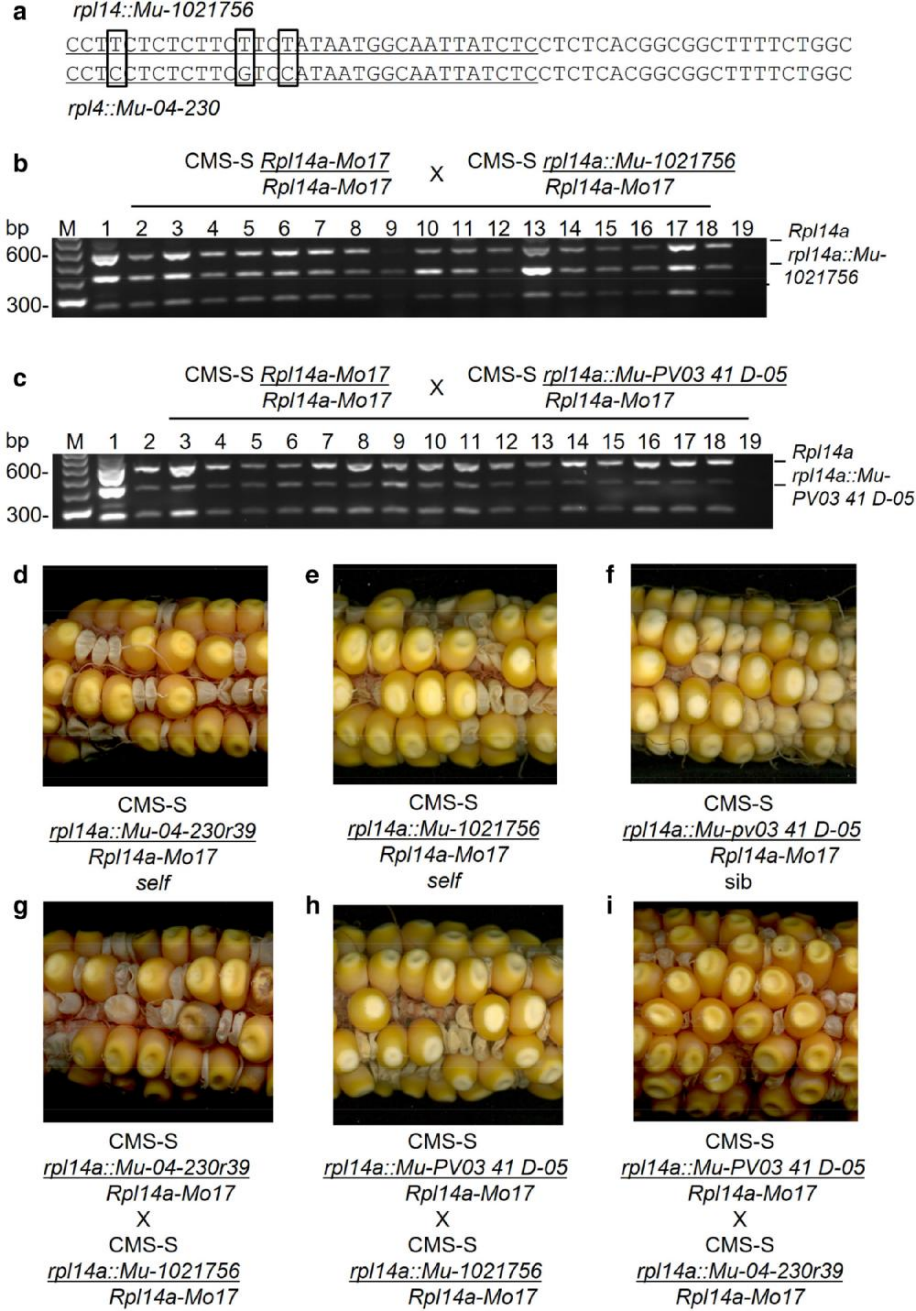
*Rpl14a::Mu-1021756* and *rpl14a::Mu-PV03 41 D-05* are independent alleles of *rpl14a::Mu-04-230.* a) Different *Mu* elements are inserted at the same position in the *rpl14a::Mu-04-230* and *rpl14a::Mu-1021756* alleles. PCR-amplified *Mu* TIR sequences (underlined) and adjacent *Rpl14*a genomic sequences are shown aligned. SNPs that distinguish the two *Mu* TIRs are boxed. (b, c) Genetic linkage between fertility restoration and *rpl14a::Mu-1021756* or *rpl14a::Mu-PV03 41 D-05*, respectively. PCR genotyping for a *Mu* TIR-*rpl14a* amplicon in a CMS-S *Rpl14a-Mo17*/*Rpl14a-Mo17* X CMS-S *Rpl14a-Mo17*/*rpl14a::Mu-1021756* or CMS-S *Rpl14a-Mo17*/*Rpl14a-Mo17* X CMS-S *Rpl14a-Mo17*/*rpl14a::Mu-PV03 41 D-05* progeny, all expected to inherit the paternal restoring allele. PCR primers were 1, 10 and 12 (Fig. 2b). M, 1 and 19 designate marker, pollen parent, and negative PCR control lanes, respectively. Lanes 2–18 show amplification products from 17 random progeny all carrying the maternal *Rpl14a-Mo17* allele and paternal *rpl14a::Mu* allele. (d–f) Segregation of mutant kernels on self or sib pollinated ears of CMS-S plants heterozygous for the *rpl14a::Mu04-230*, *rpl14a::Mu-1021756* or *rpl14a::Mu-PV03 41 D-05* mutation, respectively. Defective kernel or empty pericarp phenotypes are seen in (d) and (e), and small kernel phenotypes in (f). (g–i) Positive tests of allelism based upon mutant seed phenotypes in crosses among CMS-S plants restored by *rpl14a::Mu-04-230, rpl14a::Mu-1021756,* or *rpl14a::Mu-PV03 41 D-05.*

Independent insertions in GRMZM2G098957 were sought to confirm the *rpl14a::Mu-04-230* candidate as an *rfl* mutation. A UniformMu (McCarty *et al.* 2013) insertion in this locus (*rpl14a::Mu-1021756*) was present in maize stock UFMu-01791. Genomic DNA extracted from a plant of this stock supported PCR amplification between *Mu* TIR and *rpl14a* primers. The DNA sequence of this amplification product was compared with that of a PCR product produced on DNA extracted from an *rpl14a::Mu-04-230/Rpl14a-Mo17* plant. This comparison revealed that both plants carried a *Mu* insertion at the same genomic position. The *Mu* TIR sequences, however, differed between the two plants, demonstrating the two insertions were independently derived from two different *Mu* transposons (Fig. 3a).

The *rpl14a::Mu-1021756* insertion was genetically linked to fertility restoration and allelic to *rpl14a::Mu-04-230*. Crosses were made to introduce *rpl14a::Mu-1021756* into the CMS-S Mo17 genetic background. In a backcross 6 family, 17 of 17 randomly selected progeny inherited the *rpl14a::Mu-1021756* allele through the restored CMS-S pollen (Fig. 3b). Earlier backcross families of 17 plants sometimes included an exceptional plant that did not inherit the *rpl14a::Mu-1021756* allele, but when one such exception was grown to maturity and followed in subsequent crosses, it was found to contain a new *rfl* mutation that was not allelic to *rpl14a::Mu-1021756* based on seed phenotype. The frequency of new *rfl* mutations therefore complicates the interpretation of co-segregation studies. A backcross 4 family of 10 CMS-S *Rpl14a-Mo17/rpl14a::Mu-1021756* plants produced an average of 52.9 ± 8.6% starch-filling pollen (Table 1). Self or sib pollination of CMS-S *Rpl14a-Mo17/rpl14a::Mu-1021756* plants produced ears segregating for a range of mutant kernel phenotypes (Fig. 3e) much as seen on ears produced by CMS-S *rpl14a::Mu-04-230* plants (Figs. 2h–j and 3d). CMS-S *Rpl14a-Mo17/rpl14a::Mu-1021756* self or sib progeny ears averaged 49.1 ± 3.9% mutant kernels (Table 2). Finally, tests of allelism conducted by crossing CMS-S *Rpl14a-Mo17/rpl14a::Mu-04-230* and CMS-S *Rpl14a-Mo17/rpl14a::Mu-1021756* plants were positive, with progeny ears averaging 46.4 ± 8.8% mutant kernels (Table 2, Fig. 3g).

A second, Independent mutant *rpl14a* allele (*rpl14a::Mu-PV03 41 D-05*) was recovered through screening of the TUSC reverse genetics resource (Meeley and Briggs 1995). This insertion was located 11 bp downstream of the *rpl14a::Mu-1021756 and rpl14a::Mu-04-230* insertion site. Pollen fertility was recovered when the *rpl14a::Mu-PV03 41 D-05* allele was crossed into the CMS-S genetic background. In a backcross 1 family, 11 CMS-S *rpl14a::Mu-PV03 41 D-05/Rpl14a-Mo17* plants produced an average of 40.6 ± 16.1% starch-filled pollen (Table 1). Fertility restoration was genetically linked to *rpl14a::Mu-PV03 41 D-05*. When a CMS-S *Rpl14a-Mo17/rpl14a::Mu-PV03 41 D-05* pollen parent was crossed onto nonrestored CMS-S Mo17 seed parents, 17 of 17 random backcross 4 progeny inherited the *rpl14a::Mu-PV03 41 D-05* allele (Fig. 3c). Again earlier backcross families of 17 plants sometimes contained an exceptional plant that did not inherit the *rpl14a::Mu-PC03 41-D05* allele. Self or sib pollinations of CMS-S *rpl14a::Mu-PV03 41-D05/Rpl14a-Mo17* plants produced ears segregating for small kernels (Fig. 3f), rather than the defective kernels seen on ears segregating for *rpl14a::Mu-04-230* or *rpla4a:Mu-1021756* homozygote progeny (Fig. 3d and e), possibly indicating that *rpl14a::Mu-PV03 41-D05* is, on its own, a weak allele with respect to the mutant kernel phenotype. In tests of allelism between *rpl14a::Mu-PV03 41-D05* and *rpl14a::Mu-1021756* or *rpl14a::Mu-04-*230, however, strong mutant kernel phenotypes were observed on the resulting ears (Table 2, Fig. 3h and i) confirming a second independent allele of *rp14a::Mu-04-230*.

### *Rfl* mutant seed phenotypes

**Fig. 4.**
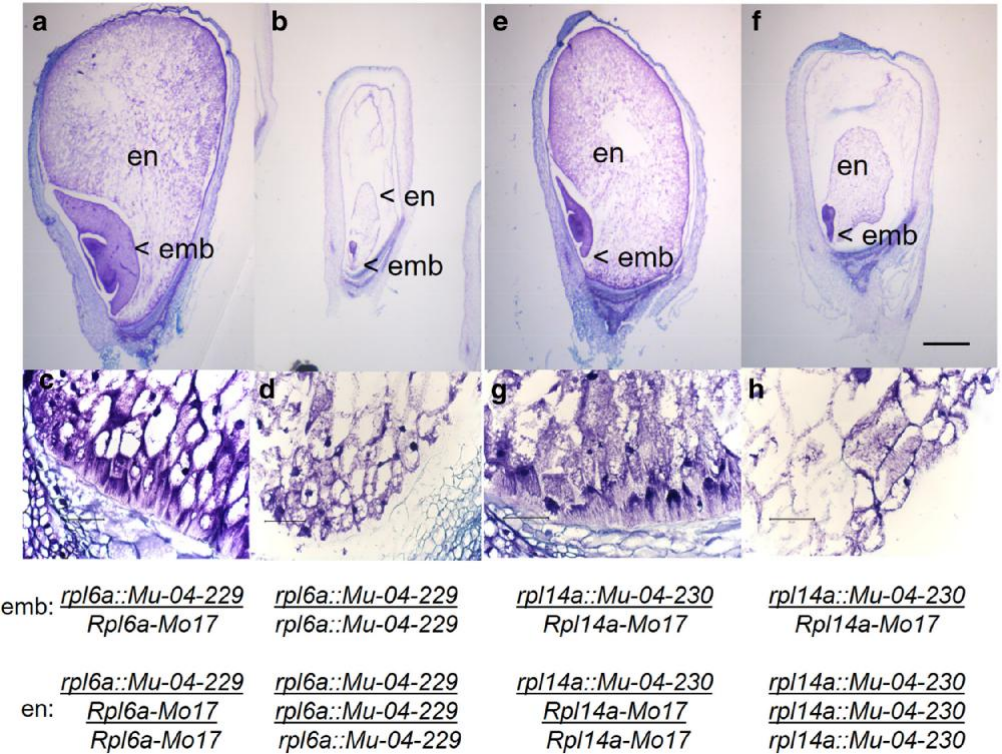
Developmental arrest of *rpl6a* and *rpl14a* homozygous mutant kernels. (a–d) Compare kernels with *rpl6a::Mu-04-229/Rpl6a-Mo17* embryo (left) to homozygous mutant *rpl6a::Mu-04-229* kernels (right) at 16 DAP. (e–h) Compare kernels with *rpl14a::Mu-04-230/Rpl1a4-Mo17* embryo (left) to homozygous mutant *rpl14a::Mu-04-230* kernels (right) at 16 DAP. emb and en indicate embryo and endosperm, respectively. (c, d, g, and h) Show structure of the BETL. Scale bars indicate 1 mm for a, b, e, and f; and 0.1 mm for c, d, g and h.

Cytological investigations of sibling mutant and nonmutant kernels were conducted to better understand the effects of the *rpl6a::Mu-04-229* or the *rpl14::Mu-04-230* mutations on seed development (Fig. 4). At 16 days after self or sib pollination of CMS-S *Rpl6a-Mo17/rpl6a::Mu-04-229* and CMS-S *Rpl14a-Mo17/rpl14a::Mu-04-230* plants, the embryos of the normal kernels had passed the coleoptile stage, the endosperm had expanded to fill the developing kernels (Fig. 4a and e), and the basal endosperm transfer layer (BETL) cells were well developed (Fig. 4c and g). In contrast, the mutant embryos had not developed past the transition stage, and mutant endosperms failed to fill the developing kernels (Fig. 4b and f). The endosperms of both mutants had a single attachment point on the adaxial side of the kernel (Fig. 4b and f), while the normal endosperms maintained contact through the base of the kernel along the BETL (Fig. 4a and e). The BETL was underdeveloped in both mutants. The invaginated cell wall structures typical of the BETL cells were less prominent and less dense in the mutants (Fig. 4d and h) compared with their normal siblings (Fig. 4c and g). These kernel phenotypes and the approximate 1:1 segregation for aborted and normal kernels (Table 2) demonstrated that mutant *rpl6a::Mu-04-229* and *rpl14a::Mu-04-230* female gametophytes developed and supported fertilization, but that mutant embryos and endosperms could not execute full kernel development.

### Expression of *rpl6a* and *rpl14a* in developing pollen

**Fig. 5.**
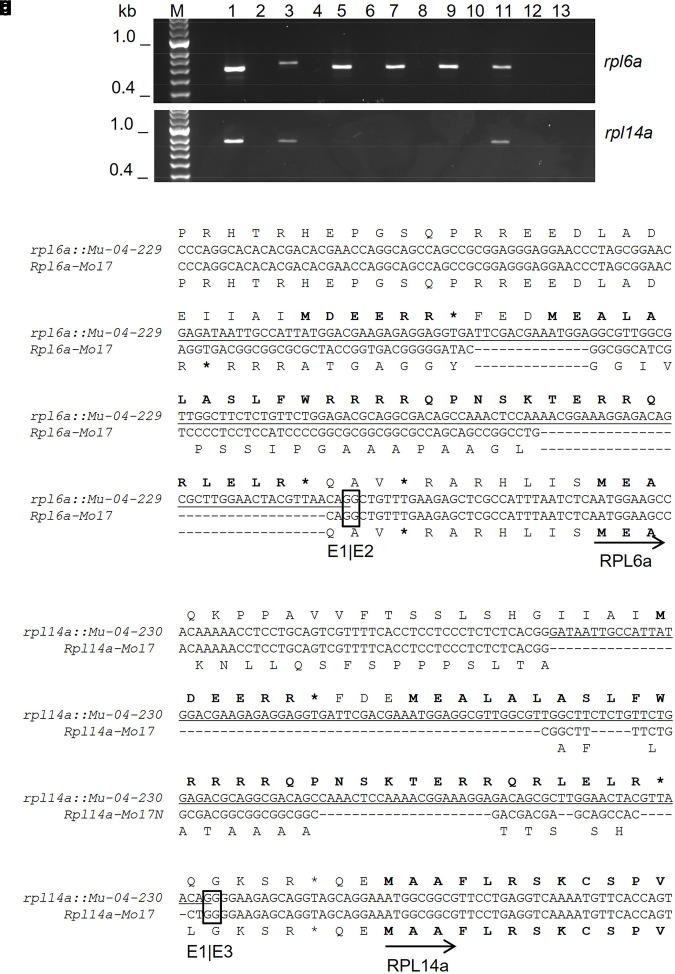
Transcripts of the *rpl6a::Mu-04-229* and *rpl4a::Mu-04-230* alleles contain *Mu* sequences and short open reading frames upstream of RPL6a and RPL14a coding sequences. (a, b) End-point reverse transcriptase PCR products of *rpl6a* and *rpl14a,* respectively, amplified with primers 3 and 8 (Fig. 1a) or 11 and 14 (Fig. 2b), respectively, and fractionated on agarose gels. Odd numbered samples contained products amplified from cDNA templates. Even numbered samples contained PCR reactions templated on cDNA synthesis controls performed in the absence of reverse transcriptase. M designates the marker lane. Pollen genotypes used for RNA extraction were N-cytoplasm Mo17 (1 and 2), CMS-S *rpl6a::Mu-04-229* (3 and 4), CMS-S *rpl14a::Mu-04-230r33* (5 and 6), CMS-S *rpl14a::Mu-04-230r39* (7 and 8), CMS-S *rpl14a::Mu-04-230r72* (9 and 10), and CMS-S Mo17 *Rf3* (11 and 12). Sample 13 contained a template-minus PCR control. c) Aligned 5′ region sequences of *Rpl6a-Mo17* and *rpl6a::Mu-04-229* cDNAs showing abnormal splicing of mutant transcripts. Translations in the frame of the RPL6a initiation codon are shown above and below the alignment. *Mu* transposon TIR sequences spliced into the mutant cDNA are underlined. The mutant transcript encodes two short open reading frames, each beginning with methionine (M) and containing an in-frame stop codon (*), upstream of and in-frame with the RPL6a initiation codon. The exon 1-exon 2 junction of the nonmutant transcript is boxed. d) Aligned 5′ region sequences of *Rpl4a-Mo17* and *rpl4a::Mu-04-230r39* cDNAs showing abnormal splicing of mutant transcripts. Weakly amplified *rpl14a::Mu-04-230* cDNAs were re-amplified for sequencing. Translations in the frame of the RPL14a initiation codon are shown above and below the alignment. *Mu* TIR sequences spliced into the mutant cDNA are underlined. The mutant transcript contains two short open reading frames, each beginning with methionine (M) and containing an in-frame stop codon (*), upstream of and in-frame with the RPL14a initiation codon. The exon 1–3 junction of the nonmutant transcript is boxed.

End-point RT-PCR and cDNA sequencing confirmed the presence of *rpl6a::Mu-04-229*, *rpl4a::Mu-04-230*, *rpl14a::Mu-1021756,* and *rpl14a::Mu-PV03 41 D-05* transcripts in rescued CMS-S pollen (Fig. 5, Supplementary Fig. 4 in Supplementary File 2). While the transcripts of *rpl14a::Mu* alleles were only weakly amplified, they were successfully re-amplified for sequencing. Sequences of the amplified cDNAs revealed that both *rpl6a::Mu-04-229* and *rpl14a::Mu-04-230* transcripts were mis-spliced to include *Mu* sequences in the 5′ UTR. In both cases, these transcripts encoded two short open reading frames, beginning with methionine initiation codons and ending with termination codons, upstream of and in the same reading frame as the downstream RPL6a or RPL14a coding sequence (Fig. 5c and d). Transcripts of the *rpl14a::Mu-1021756* and *rpl14a::Mu-PV03 41 D-05* alleles, although weakly detected, were spliced identically to *Rpl14a-Mo17* transcripts. Exon 2 was skipped in all, and there were no upstream methionine-to-termination open reading frames in-frame with RPL14a (Supplementary Fig. 4 in Supplementary File 2).

### Mitochondrial protein accumulation in pollen development

**Fig. 6.**
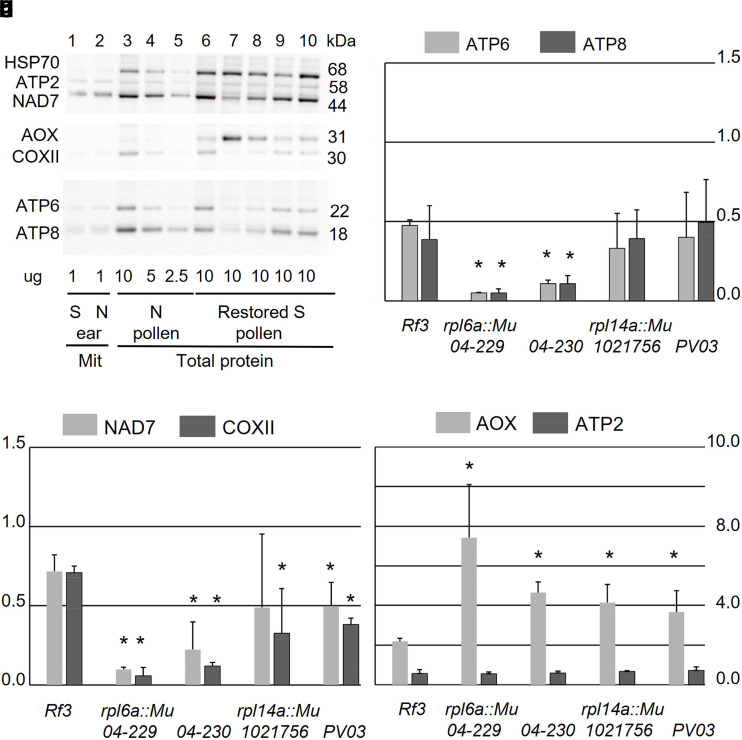
Mitochondrial protein accumulation in N-cytoplasm or restored CMS-S pollen. a) Proteins: AOX, alternative oxidase; ATP2, ATP 6, ATP8, mitochondrial ATP synthase subunits 2, 6, and 8, respectively; COXII, cytochrome oxidase subunit 2; HSP70, heat shock protein 70; and NAD7, NADH dehydrogenase subunit 7 were immunodetected in total pollen protein samples following denaturing gel electrophoresis and transfer to nitrocellulose membranes. Each blot was decorated with the HSP70 loading control and sequentially decorated with antibodies recognizing proteins of nonoverlapping size. Sample lanes contained: 1 and 2, 1 µg of protein extracted from CMS-S (S) and normal (N) cytoplasm immature ear mitochondrial pellets, respectively; 3–5, 10, 5 and 2.5 µg of total protein extracted from starch-filling, N-cytoplasm pollen, respectively; 6–10, 10 µg of total protein extracted from starch-filling, S-cytoplasm pollen restored to fertility by *Rf*3, *rpl6::Mu-04-229*, *rpl14a::Mu-04-230*; *rpl14a::Mu-1021756*, and *rpl14::Mu-PV03 41 D-05* respectively. (b–d) Abundance of proteins relative to the nuclear-encoded, mitochondria-targeted HSP70 loading controls for each pollen genotype and compared between restored S-cytoplasm starch-filling pollen and N-cytoplasm starch-filling pollen (set = 1). Error bars correspond to the standard deviation for three biological replicates. For each protein, the * symbols designate restored S-cytoplasm pollen genotypes that differ from the CMS-S *Rf3* genotype at *P* < 0.05 by a one-sided *T*-test assuming unequal variance.

Attempts to develop antibodies for the detection of RPL6a and RPL14a proteins or the CMS-S associated *orf355* gene product were unsuccessful, but antibodies to other mitochondrial proteins afforded the opportunity to study the accumulation of these proteins in the restored CMS-S pollen. Immunoblotting was used to examine the effect of pollen nuclear genotype on the abundance of the mitochondria-encoded respiratory proteins NAD7, COXII, ATP6, and ATP8. The accumulation of nuclear-encoded, mitochondria-targeted HSP70, ATP2, and AOX was also investigated (Fig. 6a). Quantified immunoblot signals, using HSP 70 as a quantitative reference, demonstrated a significant reduction of ATP6, ATP8 NAD7, and COXII proteins in CMS-S pollen rescued by *rpl6a::Mu-04-229* or *rpl14a::Mu-04-230* (Fig. 6b and c) relative to CMS-S Mo17 pollen rescued by the *Rf3* restoring allele. Mitochondria-encoded protein accumulation was, however, less affected in CMS-S pollen rescued by *rpl14a::Mu-1021756* or *rpl14a::Mu-PV03 41 D-05*. While COXII was significantly reduced in both genotypes, NAD7 was not significantly decreased in *rpl14a::Mu-1021756* pollen and ATP6 and ATP8 were not significantly reduced in either pollen genotype (Fig. 6b and c). There was no apparent influence of any *Mu* insertion allele on the abundance of nuclear-encoded, mitochondria-targeted ATP2, but CMS-S pollen rescued by each of these mutants accumulated significantly more nuclear-encoded AOX, 2–3 times that of CMS-S *Rf3* pollen (Fig. 6d).

### *Rfl* mutant pollen function

**Fig. 7.**
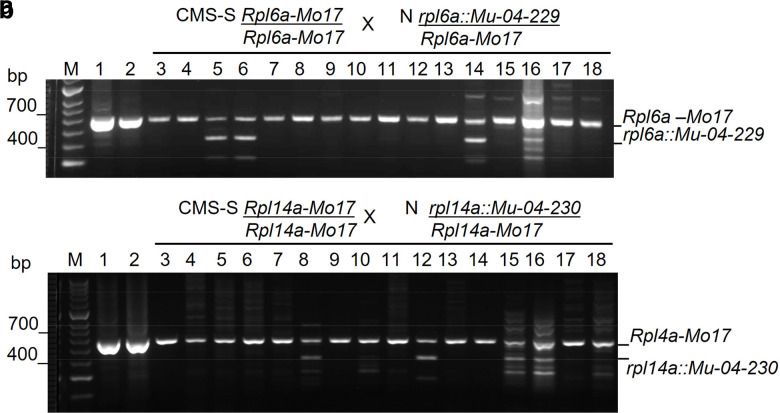
Transmission of mutant *rpl6a* and *rpl14a* alleles in N-cytoplasm pollen. a) The transmission of *rpl6a::Mu-04-229* and *Rpl6a-Mo17* alleles through N-cytoplasm pollen of a heterozygous plant assayed by genotyping progeny seedlings for mutant and nonmutant alleles. Primers 1, 3, and 5 (Fig. 1a) were used in combination. All progeny carried the *Rpl6a-Mo17* maternal allele and an *Rpl6a-Mo17* or *rpl6a::Mu-04-229* paternal allele. M designates the DNA sizing ladder. Samples 1–2 contained PCR products generated on control DNA templates extracted from Mo17 and B73 lines, respectively. Samples 3–18 were PCR products generated from 16 of 104 seedlings genotyped. b) The transmission of *rpl14a::Mu-04-230* and *Rpl14a-Mo17* alleles in N-cytoplasm pollen produced by a heterozygous plant. Primers 1, 11, and 12 (Fig. 2b) were used in combination. All progeny carried the *Rpl14a-Mo17* maternal allele and an *Rpl14a-Mo17* or *rpl14a::Mu-04-230* paternal allele. The lanes are labeled as described for (a), above, with 3–18 representing 16 of 101 seedlings genotyped.

The S-cytoplasm *rpl6a::Mu* and *rpl14a::Mu* pollen genotypes were clearly able to accomplish fertilization (Figs. 1b and 3b), whereas the CMS-S nonrestored pollen was collapsed, providing no opportunity for the functional comparison of pollen with and without the full complement of mitochondrial proteins. To investigate the effects of reduced mitochondrial protein accumulation on pollen function, N-cytoplasm Mo17 plants were fertilized with CMS-S *rpl6a::Mu-04-229* or CMS-S *rpl14a::Mu-04-230* pollen. The resulting N-cytoplasm heterozygotes, *rpl6a::Mu-04-229/Rpl6a-Mo17* and *rpl14a::Mu-04-230/Rpl14a-Mo17*, were crossed as pollen parents to nonrestored CMS-S B73 plants. Of 104 progeny seedlings genotyped for the *rpl6a::Mu-04-229/Rpl6a-Mo17* pollen parent, only 14 inherited the *rpl6a::Mu-04-229* allele. Of the 101 progeny seedlings genotyped for the *rpl14a::Mu-04-230/Rpl14a-Mo17* pollen parent, only 30 carried the *rpl14a::Mu-04-230* insertion allele (Fig. 7a and b). Both progenies deviated significantly from the expected 1:1 ratio for inheritance of the two paternal alleles. The Yates-corrected *X*^2^ and *P* values for this ratio were 54.1, *P* = 0 for the *rpl6a::Mu-04-229* family and 15.8, *P* = 0 for the *rpl14a::Mu-04-230* family. This demonstrated a significant bias against transmission of the *rpl6a::Mu-04-229* or the *rpl14a::Mu-04-230* allele in N-cytoplasm pollen.

## Discussion

### Paradigms of fertility restoration

Our findings corroborate a loss-of-function model for fertility restoration in CMS-S maize (Wen *et al.* 2003; Gabay-Laughnan *et al.* 2018) and a novel fertility restoration mechanism more broadly compromising mitochondrial gene expression as compared with those characterized so far (Chen and Liu 2014; Anisimova 2020; Kubo *et al.* 2020; Gautam *et al.* 2023). The independent *rpl14a::Mu-1021756* and *rpl14a::Mu-PV03 D-05* alleles confirmed the *rpl14a::Mu-04-230* candidate as rfl*-04-230. Although the rfl*04-229 candidate *rpl6a::Mu-04-229* could not be verified by an independent allele, the many parallels with rfl*-04-230 provide strong support for this restorer mutant candidate. A related example is a reduced-expression allele of *RMS* that restores fertility in the gametophytic CW CMS of rice (Fujii and Toriyama 2009; Suketomo *et al.* 2020). Here, however, the reduced-expression allele has no effect on mitochondrial gene expression. Rather the functional *RMS* allele encodes a mitochondria-targeted protein required to condition pollen sterility (Toriyama 2021). In contrast to loss-of-function restorers, most CMS restorers are gain-of-function alleles encoding mitochondria-targeted PPR proteins (Chen and Liu 2014; Anisimova 2020; Kubo *et al.* 2020; Gautam *et al.* 2023). Phylogenetic analysis of genes predicting mitochondria-targeted PPR proteins revealed that restorer *PPR* and *Rf-PPR-like* genes are a distinct, rapidly evolving clade of the *PPR* gene family, consistent with the gain of new functions that silence specific mitochondrial CMS gene targets (Fujii *et al.* 2011; Dahan and Mireau 2013). In keeping with this model, the CMS-S *Rf3* restoring allele is a dominant, gain-of-function allele (Kamps *et al.* 1996) that predicts an 814-amino acid, mitochondria-targeted PPR protein. This coding sequence is absent in nonrestoring maize lines but, when present, lies adjacent to a related PPR protein gene found in all maize lines examined (Qin *et al.* 2021). Fertility restoration by *Rf3* is associated with loss of RNA editing, increased internal cleavage and decreased abundance of *orf355* transcripts (Qin *et al.* 2021).

Despite identification of the *rpl6a* candidate and *rpl14a* restorer alleles, questions remain regarding the exact mechanism of fertility restoration. Ectopic expression studies associate ORF355 with respiratory complex I deficiency (Xiao *et al.* 2023) and CMS-S pollen collapse (Xiao *et al.* 2020). A straight-forward model for fertility restoration is that reducing mitochondrial protein synthesis limits accumulation of the ORF355 protein, thereby preventing the cascade of events (Chamusco *et al.* 2022) leading to pollen collapse. A direct protein assay for ORF355 is, however, still lacking and is needed to confirm this model.

The variable effects of the different *rpl6a* and *rpl14a* alleles on the accumulation of mitochondrial translation products suggest an alternative model based on the increased accumulation of nuclear-encoded, mitochondria-targeted AOX. The AOX nonphosphorylating branch of the plant mitochondrial respiratory pathway is induced in response to conditions limiting conventional mitochondrial respiratory electron transfer. AOX reduces the production of damaging reactive oxygen molecules and is an important component of plant stress response and signaling pathways (Rasmusson *et al.* 2009; Van Aken *et al*. 2009; Saha *et al.* 2016). The upstream reading frames with in-frame stop codons spliced into the *rpl6a::Mu-04-229* and *rpl14a::Mu-04-230* transcripts might condition stronger mutant alleles accounting for greater loss of mitochondria-encoded respiratory proteins in these pollen genotypes compared with the relatively weaker effects of the *rpl14a::Mu-1021756* and *rpl14a::Mu-PV03 41 D-05* alleles. Nevertheless, increases in AOX abundance are comparable in pollen restored by each of the four mutations. AOX was barely immunodetected in N-cytoplasm pollen and was increased about 2-fold in CMS-S *Rf3* pollen compared with N-cytoplasm pollen. While the relative contributions of the S-cytoplasm and the *Rf3* allele cannot be distinguished in that comparison, AOX abundance in restored CMS-S *rpl6a* or CMS-S *rpl14a* pollen was 2-fold greater than in CMS-S *Rf3* pollen. Over expressing AOX in developing pollen possibly constitutes a mechanism of fertility restoration in CMS-S maize and appropriate AOX over expression with respect to timing and tissue might provide a strategy for the rescue of pollen function in some of the other CMS systems.

### Genetics of plant mitochondrial ribosome biogenesis and function

Plant mitochondrial ribosomes are assembled from nuclear and mitochondria- encoded components (Tomal *et al.* 2019). While the 26S, 18S, and 5S ribosomal RNAs are always encoded by the mitochondrial genome, protein components are contributed by both genomes. The coding location of some mitochondrial ribosomal proteins varies across plant species, but *rpl6* and *rpl14* are, so far, strictly nuclear-encoded genes (Sugiyama *et al.* 2005; Kubo and Newton 2008). Proteomic studies of Arabidopsis mitochondrial ribosomes identified 92 proteins. In addition to canonical large and small subunit core proteins, protein maturases and PPR proteins were identified as bona fide components having roles in ribosome biogenesis and function (Rugen *et al.* 2019; Waltz *et al.* 2019; Waltz *et al.* 2020).

Mutations that disrupt plant mitochondrial ribosome biogenesis condition different phenotypes depending upon the contribution of the gene to mitochondrial translation and the presence or absence of functional paralogs. Cross talk between the two genomes contributing to mitochondrial translation is evident even in mutations having subtle effects upon system components. For example, down-regulating the mRNA for the nuclear-encoded mitochondrial RPS10 in Arabidopsis does not alter the overall abundance of the RPS10 protein but nevertheless causes a translational imbalance within the mitochondria, favoring the translation of the mitochondria-encoded ribosomal proteins over respiratory subunits (Kwasniak *et al.* 2013). A battery of effects on mitochondrial RNA abundance, intron splicing, and ribosome engagement observed in *RPS10* mutant leaves points to the complexities of plant mitochondrial gene expression system and, potentially, roles for ribosomal proteins outside of translation (Kwasniak-Owczrek *et al*. 2019).

Serious defects in plant mitochondrial ribosome function generally condition lethality. The maize nonchromosomal stripe (NCS) mutants NCS3 and NCS4 delete the mitochondrial gene encoding RPS3. These mutations severely compromise mitochondrial function such that they are only viable in heteroplasmic combination with nonmutant mitochondrial genomes (Hunt and Newton 1991; Newton *et al.* 1996). The homozygous-lethal *rpl6a::Mu* and *rpl14a::Mu* mutations are consistent with essential contributions of RPL6 and RPL14 to mitochondrial translation. RPL14 is a conserved component of the ribosomal large subunit. Human mitochondrial RPL14 (MRPL14) interacts with a DUF143 protein (C7orf30) that functions as a ribosome assembly factor (Fung *et al.* 2013). C7orf30 is related to the maize IOJAP protein, required for plastid ribosome stability (Walbot and Coe 1979; Han *et al.* 1992) and to a mitochondria-targeted IOJAP paralog predicted by GRMZM6G577626_T01. In human cells, depletion of MRPL14 or C7orf30 causes the loss of mitochondrial monosomes, a mitochondrial translation defect and a 25–75% decrease in the accumulation of respiratory complex subunits (Rorbach *et al.* 2012; Wanschers *et al.* 2012; Fung *et al.* 2013). RPL6 is also a conserved protein essential to ribosome function. In *Bacillus subtilis*, the addition of RPL6 to the large subunit is chaperoned by the conserved GTPase assembly factor RbgA and is required for the subsequent incorporation of RPL16 for formation of a functional large subunit (Lancaster *et al.* 2008; Gulati *et al.* 2014). In yeast, mutations in the nuclear gene encoding mitochondrial RPL6 cause the loss of mitochondrial translation and a respiratory deficient phenotype (Harrer *et al.* 1993).

In maize, the *rpl6a::Mu* and *rpl14a::Mu* alleles share many features with mutations in the *dek44* gene, which encodes a mitochondrial ribosomal protein L9 (Qi *et al.* 2019). Mutations at all three loci condition inviable kernels having similar morphological phenotypes, discussed below. Protein phenotypes of *dek44* mutant kernels and the *rpl6a::Mu* and *rpl14a::Mu* pollen include the loss of mitochondria-encoded respiratory proteins and increased accumulation of nuclear-encoded, mitochondria-targeted AOX. AOX up-regulation in the ribosomal protein mutants is, however, apparently insufficient to rescue seed development.

### Mitochondrial requirements for seed development

Self or sib pollinations of CMS-S plants heterozygous for the *rpl6a* and *rpl14a* mutations produced ears approximating a 1:1 segregation ratio of mutant and normal kernels. This demonstrated that these mutations did not have significant effects on development of the haploid female gametophyte. This contrasts with observations in Arabidopsis, where mutations in several nuclear genes encoding mitochondrial ribosomal proteins block development of the female gametophyte (Robles and Quesada 2017). Examples include mutations in the *NUCLEAR FUSION DEFECTIVE1* (*NFD1*) and *NFD3* genes, encoding mitochondrial RPL21 and RPS11, respectively (Portereiko *et al.* 2006), as well as mutations decreasing accumulation of mitochondrial RPS9 (Lu *et al.* 2017). These mutations block fusion (karyogamy) of the polar nuclei that form the central cell nucleus and, ultimately, the endosperm. Consequently, they do not transmit effectively through the seed (female) parent. There is no current evidence of a stringent requirement for mitochondrial ribosomal proteins in the maize female gametophyte. It is unknown whether this paradox is due to developmental variation in the timing of karyogamy, metabolic differences in gametophytes of the two species, or specific roles of the genes identified by mutation to date.

Despite the lack of a mutant phenotype at the haploid female gametophyte stage, *rpl6a::Mu-04-229* and *rpl14a::Mu-04-230* and a large number of additional maize mutants demonstrate the critical importance of mitochondrial function to subsequent stages of maize seed development. Genes encoding proteins with roles in mitochondrial gene expression comprise the majority of cloned *defective kernel* (*dek*), *small kernel* (*smk*) and *empty pericarp* (*emp*) loci of maize. Almost all of these encode PPR proteins required for mitochondrial intron splicing or RNA editing events (Dai *et al.* 2021). When homozygous, the *dek* and *emp* mutants condition kernel phenotypes much like *dek44*, *rpl6a::Mu-04-229* and *rpl14a::Mu-04-230*, having under developed embryos and endosperms, along with a rudimentary BETL (Dai *et al.* 2021) lacking the extensive membrane system that normally develops and supports the energy dependent transport of solutes between maternal tissue and developing seed (Gao *et al.* 1998).

The extent to which these seed development mutants might also function as CMS-S fertility restoration mutants is an interesting question. The answer depends upon the expression patterns of genes in question, whether there are paralogs with duplicate or overlapping functions, and the mutation's effects on mitochondrial processes. The DEK44 protein, for example, does not accumulate in maize tassels (Qi *et al.* 2019) so the *dek44* mutant is not predicted to effect fertility restoration. Several mutants compromise editing or splicing of mitochondrial transcripts encoding ribosomal proteins (Gutiérrez-Marcos *et al.* 2007; Manavski *et al*. 2012; Liu *et al.* 2013; Yang *et al.* 2017; Chen *et al.* 2019; Wang *et al.* 2019; Dai *et al.* 2020; Ren *et al.* 2020) leading to defects in mitochondrial translation. In addition, the upregulated expression of AOX is commonly observed in homozygous *emp*, *smk,* and *dek* mutant kernels (Chen *et al.* 2019; Wang *et al.* 2019; Dai *et al.* 2020; Ren *et al.* 2020). Given these features, some overlap between the *dek-smk-emp* and *rfl* mutant classes seems likely. The overlap between seed-lethal and restorer-of-fertility mutants will better define mitochondrial requirements for seed and pollen development as well as nuclear gene contributions to mitochondrial function overall.

### Mitochondrial requirements in pollen function

The role of mitochondria in CMS (Linke and Borner 2005; Carlsson *et al.* 2008; Horn *et al.* 2014), increasing numbers of mitochondria during pollen development (Lee and Warmke 1979) and high rates of respiration during pollen tube germination (Tadege and Kuhlemeier 1997) all point toward the importance of mitochondrial respiration in pollen development and function (Rounds *et al.* 2011; Colaço *et al.* 2012). Mitochondrial biogenesis is temporally regulated in maize pollen development. The abundance of respiratory proteins is very low in uninucleate microspores and significantly increased during the bi-cellular stage following the microspore mitosis (Chamusco *et al.* 2022). Mature, tri-cellular pollen contains no mitochondrial transcripts (Wen and Chase 1999), and so mitochondrial translation is active in the developmental window between the uninucleate microspore and mature pollen stages. Despite compromised mitochondrial biogenesis, CMS-S *rpl6a::Mu-04-229* and CMS-*S rpl14a::Mu-04-230* pollen genotypes are fully capable of accomplishing fertilization. In N-cytoplasm, however, the *rpl6a::Mu-04-229* and *rpl14a::Mu-04-230* pollen genotypes did not compete effectively with nonmutant pollen. This is likely due to limited capacity for respiration and ATP synthesis conditioned by lack of respiratory protein subunits. This effect is also apparent in Arabidopsis, where the mutations in genes encoding mitochondrial ribosomal proteins also show reduced transmission through male gametophytes (Portereiko *et al.* 2006; Lu *et al.* 2017). The strong bias against transmission of the *rpl6a::Mu-04-229* allele in N-cytoplasm pollen likely contributed to problems recovering an independent mutant allele. Possibly CRISPR/Cas9 mutagenesis (Hernandes-Lopes *et al.* 2023) performed in a CMS-S background would yield confirming independent alleles for the *rpl6a* candidate restorer.

Fertilization by CMS-S *rfl* mutant pollen does, however, contribute evidence of metabolic flexibility in maize pollen function as also seen in other species. Despite the presence of numerous mitochondria organized in a longitudinal gradient of organelles (Colaço *et al.* 2012), the lily pollen tube adapts to and continues growth in the presence of mitochondrial respiratory inhibitors. This metabolic adaptation includes the activation of a gamma-aminobutyric acid shunt from the TCA cycle and a pyruvate decarboxylase—alcohol dehydrogenase fermentation pathway (Rounds *et al.* 2010; Obermeyer *et al.* 2013). In petunia and tobacco pollen, aerobic fermentation through a pyruvate dehydrogenase bypass comprised of pyruvate decarboxylase and aldehyde dehydrogenase is active during normal pollen tube growth (Mellema *et al.* 2002; Gass *et al.* 2005). This bypass has been hypothesized to support pollen fertility in maize (Tadege *et al.* 1999), but it is not active in pollen of Arabidopsis (Liu *et al.* 2022). The importance of both cytosolic and plastid glycolytic pathways has been noted in Arabidopsis pollen tube growth (Selinski and Scheibe 2014; Liu *et al.* 2022). The metabolic pathways supporting pollen maturation and function in *rfl* pollen of CMS-S maize require further investigation and will provide additional insights into the mitochondrial requirements and alternative metabolic strategies that support pollen development and fertility.

### Material and data availability

The mutants described in this work, backcrossed into the CMS-S Mo17 genetic background for four or more generations, are available from the Maize Genetics Cooperation Stock Center or from the corresponding author. All data are contained in the manuscript and Supplementary materials. Supplementary File 1 describes the genetic materials, PCR primers, protein targeting prediction results, and pollen fertility data used to identify the *rfl* candidate loci. Supplementary File 2 describes the genetic crosses used to develop materials for *Mu* Illumina, sequences of the *rpl6::Mu* insertion region, sequences of the *rpl14a::Mu* insertion region, and full-length cDNA sequences of *rpl14a::Mu-1021756* and *rpl14a::Mu-Pv03 41 D-05* alleles.

Supplemental material available at G3 online.

Note added in proof: Maize gene models Zm00001eb104220 and Zm00001eb426610 are designated *rpl14a* and *rpl14b* in the Maize Genetics and Genomics Database (https://www.maizegdb.org/). These genes predict cytosolic ribosomal proteins that have no significant amino acid similarity with GRMZM2G098957 / Zm00001d041322. GRMZM2G098957 / Zm00001d041322 will therefore carry the database designator *rpl14c*.

## Supplementary Material

jkae201_Supplementary_Data
