# Supplementary material for: Mutations in nuclear genes encoding mitochondrial ribosome proteins restore pollen fertility in S male-sterile maize
Source: G3 (Bethesda). 2024 Aug 20;14(10):jkae201. doi: 10.1093/g3journal/jkae201 (PMC12117434; doi:10.1093/g3journal/jkae201)
Supplement: jkae201_Supplementary_Data [file jkae201_Supplementary_Data.zip › File_S1_G3-2024-405213.pdf]

**Table S1 Genetic Materials**

| Stock | Cytoplasm | Nucleus |
|---|---|---|
| Mo17-S | CMS-S | Mo17 |
| Mo17-N | N[a] | Mo17 |
| B73-S | CMS-S | B73 |
| B73-N | N | B73 |
| ccB73-N *Mu*-on[b] | N | ccB73 *Mu*-on |
| ccB73-S *Mu*-on | CMS-S | ccB73 *Mu*-on |
| Mo17-S *Rf3-CE1/rf3-Mo17* | CMS-S | Mo17[c] |
| Mo17-S *rfl*-04-229/Rfl*-04-229-Mo17* | CMS-S | Mo17[d] |
| Mo17-S *rfl*-04-230/Rfl*-04-230-Mo17* | CMS-S | Mo17[e] |
| UFMu-01791 | N | W22 UniformMu |
| PV03 41 D-05 | N | TUSC PV03 |

[a] Normal cytoplasm that does not induce pollen sterility

[b] color-converted, transposon *Mu*-active derivative of the B73 inbred line

[c] *Rf3-CE1* back-crossed to Mo17 for 17 generations

[d] *rfl*04-229* pollen crossed onto Mo17-S for three or more generations

[e] *rfl*04-230* pollen crossed onto Mo17-S for two or more generations

**Table S2 Oligonucleotide primers**

| Name | Sequence 5'-3' | Location and orientation |
|---|---|---|
| 1 | GCCTCCATTTCGTCGAATCC | *Mu* TIR |
| 2 | CATCGGTGATCTAGTAGTAGTACC | *rpl6a* (GRMZM2G080608) 5' Flank F[a] |
| 3 | AACCAAGAGCCAACAGAG | *rpl6a* 5' UTR[b] F |
| 4 | AGGCACACACGACACGAACC | *rpl6a* 5' UTR F |
| 5 | ATGGCTTCAGGTTCAGGCTTTCA | *rpl6a* intron 1 R[c] |
| 6 | TGGCGAGCTCTTCAAACAGC | *rpl6a* Exon 2 R |
| 7 | CACAACACAGCAGACCCCAGATCCAG | *rpl6a* Exon 2 R |
| 8 | CAGCCGTACAGGATAGTTTG | *rpl6a* 3'UTR R |
| 9 | GAGCGGTGGGAGCAGCAT | *alb3a* (GRMZM5G839422) exon1 R |
| 10 | CTGACCGCTTTCATCCACGCAT | *rpl14a* (GRMZM2G098957) 5' flank F |
| 11 | ACAAAAACCTCCTGCAGTCG | *rpl14a* 5' UTR F |
| 12 | ATGCAACCCCTGCCGAACAA | *rpl14a* exon 2 R |
| 13 | GGGAAGAAAGGAGCAAGGCT | *rpl14a* exon 5 F |
| 14 | TGGACGAGCAATGTCATC | *rpl14a* 3' UTR R |
| 15 | GCCTTGCTCTTTCTTCCC | *rpl14a* exon 5 R |
| 16 | TCTTCGTGAAGACCCTGACC | *Ubi1* (GRMZM2G409726) exon 2 F |
| 17 | GACGACGCAGGCACATCG | *Ubi1* exon 2 R |

[a] F, Forward orientation with respect to the gene

[b] UTR, untranslated region

[c]R, Reverse complement with respect to the gene

**Table S3 *rfl*-04-229* candidate *rpl6a* gene and paralogs**

| Gene Model[a] | Gene Location[b] | Transcript[a] | Predicted Protein Length[a] | Predicted Protein Location iPSORT[c] | Predicted Protein Location TargetP[d] |
|---|---|---|---|---|---|
| GRMZM2G080608[e] (Zm00001d051422) | 4:158027318 - 158027632 | T001 | 104 | O | 0.913 (O) 0.037 (M) |
| GRMZM2G86788 (Zm00001d029201) | 1: 61426173 - 61429704 | T001 | 226 | P | 0.996 (P) 0.003 (O) |
| GRMZM2G86788 (Zm00001d029201) | 1:61426173 - 61427847 | T002 | 119 | M | 0.982 (O) 0.017 (M) |
| GRMZM2G86788 (Zm00001d029201) | 1: 61427987 - 61429704 | T003 | 115 | P | 0.989 (P) 0.009 (O) |
| GRMZM2G170870 (Zm00001d047462) | 9:134475499 134479362 | T001 | 226 | P | 0.999 (P) |

[a] Gene models, transcripts and predicted protein lengths in amino acids from http://www.gramene.org/, release 53, accessed 7/16/2017

[b] Gene coordinates updated from http://www.gramene.org/, release 66, accessed 9/6/2023, except for GRMZM2G080608f (Zm00001d051422) which is not present in recent gramene releases

[c] Protein locations: M, mitochondrial; P, plastid; O, other predicted by the iPSORT WWW Service http://ipsort.hgc.jp/#predict (Bannai *et al.* 2002), accessed 12/02/2014

[d] Protein locations predicted by the TargetP 2.0 server https://services.healthtech.dtu.dk/services/TargetP-2.0/ (Almagro Armenteros *et al.* 2019), accessed 9/6/2023

[e] *rfl*-04-229* candidate locus identified by *Mu* Illumina

**Table S4 *rfl*-04-230* candidate *oxa1/alb3/yidC* gene**

| Gene Model[a] | Gene Location[b] | Transcript[a] | Predicted Protein Length[a] | Predicted Protein Location iPSORT[c] | Predicted Protein Location TargetP[d] |
|---|---|---|---|---|---|
| GRMZM5G839422[e] (Zm00001d03985) | 3: 17628283 - 17629264 | T001 | 455 | M | 0.981 (P) 0.002 (M) |

[a] Gene model, transcript and predicted protein length in amino acids from http://www.gramene.org/, release 53, accessed 7/17/2017

[b] Gene coordinates updated from http://www.gramene.org/, release 66, accessed 9/6/2023

[c] Protein locations M, mitochondrial; P, plastid; O, other predicted by the iPSORT WWW Service http://ipsort.hgc.jp/#predict (Bannai *et al.* 2002), accessed 12/02/2014

[d] Protein locations predicted by the TargetP 2.0 server https://services.healthtech.dtu.dk/services/TargetP-2.0/ (Almagro Armenteros *et al.* 2019), accessed 9/6/2023

[e] *rfl*-04-230* candidate locus identified by *Mu* Illumina

**Table S5 *rfl*-04-230* candidate *rpl14a* gene and paralogs**

| Gene Model[a] | Gene Location[b] | Transcript[a] | Predicted Protein Length[a] | Predicted Protein Location iPSORT[c] | Predicted Protein Location TargetP[d] |
|---|---|---|---|---|---|
| GRMZM2G098957[e] (Zm00001d041322) | 3:113440225 - 113446068 | T001 | 170 | M | 0.884 (M) 0.221 (O) |
| GRMZM2G098957[e] (Zm00001d041322) | 3:113440225 - 113446061 | T002 | 170 | M | 0.608 (M) 0.381 (P) |
| GRMZM2G098957[e] (Zm00001d041322) | 3:113440227 - 113446061 | T003 | 117 | M | 0.962 (O) 0.035 (M) |
| GRMZM5G804776 | Pt[f]:79,038 - 79,409 | T001 | 123 | N/A | N/A |

[a] Gene model, transcript and predicted protein length in amino acids from http://www.gramene.org/, release 53, accessed 7/16/2017

[b] Gene coordinates updated from http://www.gramene.org/, release 66, accessed 9/6/2023

[c] Protein locations: M, mitochondrial; P, plastid; O, other predicted by the iPSORT WWW Service http://ipsort.hgc.jp/#predict (Bannai *et al.* 2002), accessed 12/02/2014 and 7/16/2017

[d] Protein locations predicted by the TargetP 2.0 server https://services.healthtech.dtu.dk/services/TargetP-2.0/ (Almagro Armenteros *et al.* 2019), accessed 9/6/2023

[e] *rfl*-04-229* candidate locus identified by *Mu* Illumina

[f] Pt, plastid genome

**Table S6 Pollen phenotypes of CMS-S plants genotyped at the *rpl14a* and *alb3a* candidate loci**

| Cytoplasm | Nuclear genotype | Pollen phenotype | |
|---|---|---|---|
| | | N[a] | % normal[b] |
| CMS-S | *alb3a::Mu-04-230 rpl14a::Mu-04-230/ Alb3a-Mo17 Rpl14a-Mo17* | 5 | 63.4 ± 4.0 |
| CMS-S | *Alb3a::Mo17 rpl14a::Mu-04-230/ Alb3a-Mo17 Rpl14a-Mo17* | 3 | 49.2 ± 4.0 |
| CMS-S | *alb3a::Mu-04-230 Rpl14a-Mo17/ Alb3a-Mo17 Rpl14a-Mo17* | 3 | 19.7 ± 3.9 |
| CMS-S | *Alb3a::Mu-04-230 Rpl14a::Mu-04-230/ Alb3a-Mo17 Rpl14a-Mo17* | 5 | 1.5 ± 1.5 |

[a] n, the number of plants that were phenotyped

[b] The mean % of normal pollen (± standard deviation) per plant, was determined by counting the number of normal (starch-filled) and aborted (empty, collapsed) pollen grains in samples of at least 400 grains taken from individual plants. Standard deviations were calculated at http://www.mathportal.org/calculators/statistics-calculator/standard-deviation-calculator.php (accessed 8/12/2015).
