# Supplementary material for: Mutations in nuclear genes encoding mitochondrial ribosome proteins restore pollen fertility in S male-sterile maize
Source: G3 (Bethesda). 2024 Aug 20;14(10):jkae201. doi: 10.1093/g3journal/jkae201 (PMC12117434; doi:10.1093/g3journal/jkae201)
Supplement: jkae201_Supplementary_Data [file jkae201_Supplementary_Data.zip › File_S2_G3-2024-405213.pdf]

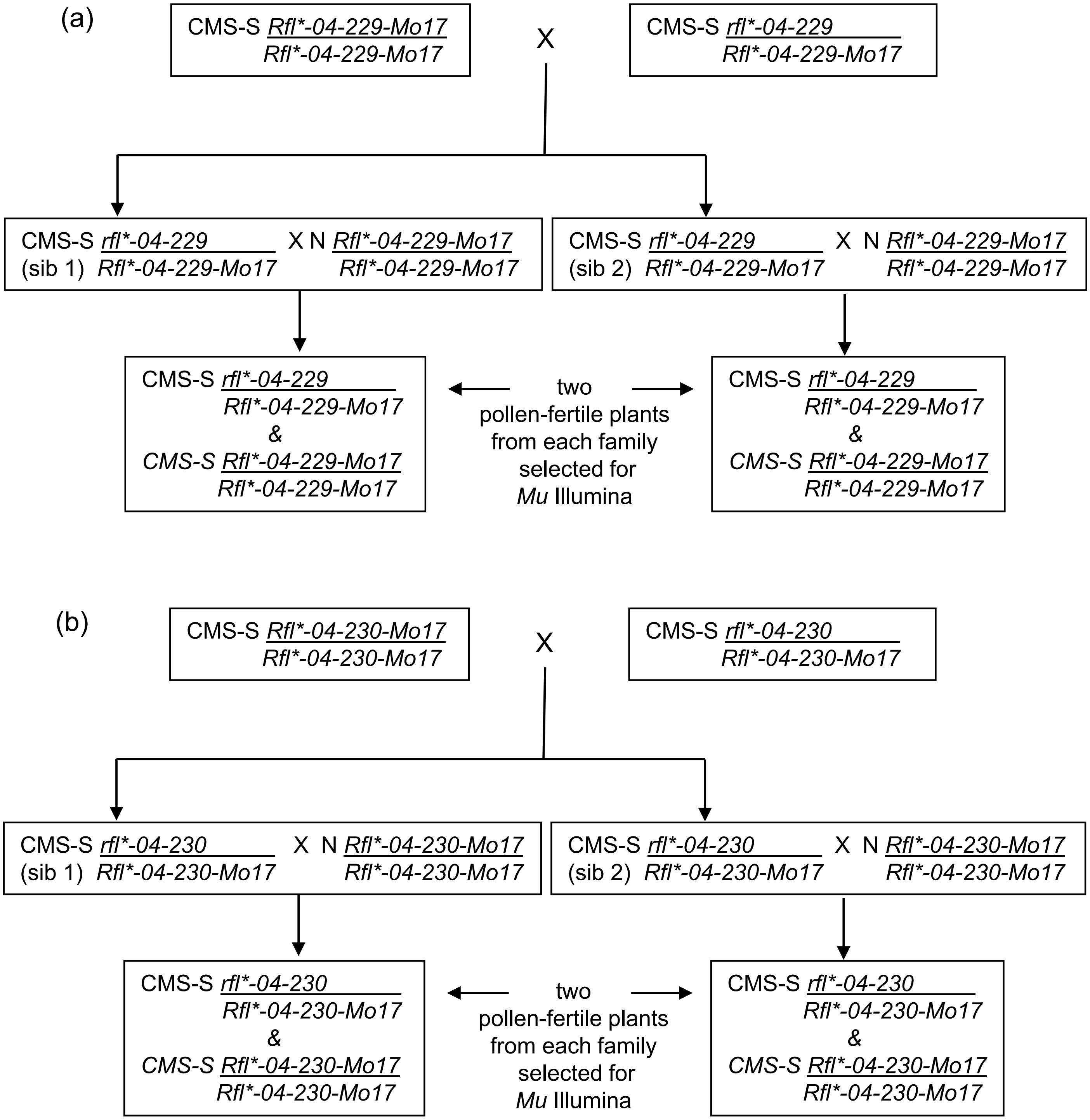

Figure S1. Genetic crosses generating pairs of *rfl** cousins for *Mu* Illumina. Identical crossing schemes were used to develop CMS-S plants that were restored by *rfl*-04-229* (a) or *rfl*-04-230* (b) for the *Mu* Illumina analysis. Each restoring (*rfl**) allele was first combined with its corresponding non-mutant, non-restoring allele from Mo17. Pairs of sibling CMS-S plants heterozygous for the *rfl** allele and its corresponding Mo17 non-mutant, non-restoring allele were then pollinated with normal (N) -cytoplasm Mo17 pollen. This created two families of CMS-S plants segregating for the *rfl** allele. Two pollen-fertile plants from each family were selected for *Mu* Illumina library construction.

```
Rpl6a-B73              T----GCGGAGGGAGGAACCCTAGCGGAACAGGTGACGGCGGCGCGCTAC
Rpl6a-ccB73 Mu-on      T----GCGGAGGGAGGAACCCTAGCGGAACAGGTGACGGCGGCGCGCTAC
Rpl6a-Mo17             CAGCCGCGGAGGGAGGAACCCTAGCGGAACAGGTGACGGCGGCGCGCTAC
rpl6a::Mu-04-229       GTCCATAATGGCAATTATCTCTAGCGGAACAGGTGACGGCGGCGCGCTAC
                                           ******************************

Rpl6a-B73              CGGTGACGGGGGATACGGCGGCATCGTCCCCTCCTCCATCCCCGGCGCGG
Rpl6a-ccB73 Mu-on      CGGTGACGGGGGATACGGCGGCATCGTCCCCTCCTCCATCCCCGGCGCGG
Rpl6a-Mo17             CGGTGACGGGGGATACGGCGGCATCGTCCCCTCCTCCATCCCCGGCGCGG
rpl6a::Mu-04-229       CGGTGACGGGGGATACGGCGGCATCGTCCCCTCCTCCATCCCCGGCGCGG
                       **************************************************

Rpl6a-B73              CGGCGCCAGCAGCCGGCCTGCAGGTACGGTCTCCAGTCTCCAGAATCCAG
Rpl6a-ccB73 Mu-on      CGGCGCCAGCAGCCGGCCTGCGGGTACGGTCTCCAGTCTCCAGAATCCAG
Rpl6a-Mo17             CGGCGCCAGCAGCCGGCCTGCAGGTACGGCCTCCAGCCTCCAGAATCCAG
rpl6a::Mu-04-229       CGGCGCCAGCAGCCGGCCTGCAGGTACGGCCTCCAGCCTCCAGAATCCAG
                       ********************* ******* ****** *************

Rpl6a-B73              ATTCAGAGCTTGTCCCTGTTTTCTGGTGTGCTCTGCGTATGGCTGTTGGC
Rpl6a-ccB73 Mu-on      ATTCAGAGCTTGTCCCTGTTTTCTGGTGTGCTCTGCGTATGGCTGTTGGC
Rpl6a-Mo17             ATTCAGAGCTTGTCCCTGTTTTCTGGTGTGCTCTGCGTATGGCTGTTGGC
rpl6a::Mu-04-229       ATTCAGAGCTTGTCCCTGTTTTCTGGTGTGCTCTGCGTATGGCTGTTGGC
                       **************************************************

Rpl6a-B73              CTGTTGCTTCCCATGTCCATGTTTGTTTGTTTTCAGTTTTACTTTGATGC
Rpl6a-ccB73 Mu-on      CTGTTGCTTCCCATGTCCATGTTTGTTTGTTTTCAGTTTTACTTTGATGC
Rpl6a-Mo17             CTGTTGCTTCCCATGTCCATGTTTGTTTGTTTTCAGTTTTACTTAGATGC
rpl6a::Mu-04-229       CTGTTGCTTCCCATGTCCATGTTTGTTTGTTTTCAGTTTTACTTAGATGC
                       ******************************************** *****

Rpl6a-B73              TGAAAGCCTGAAACCTTCCAGTGATTATTTGCTTCATGTTGTAGTATGGC
Rpl6a-ccB73 Mu-on      TGAAAGCCTGAAACCTTCCAGTGATTATTTGCTTCATGTTGTAGTATGGC
Rpl6a-Mo17             TGAAAGCCTGAAACCTTCCAGTGATTATTTGCTTCATGTTGTAGTATGGC
rpl6a::Mu-04-229       TGAAAGCCTGAAACCTTCCAGTGATTATTTGCTTCATGTTGTAGTATGGC
                       **************************************************
```

Figure S2. The *rpl6a::Mu-04-229* allele arose by insertion into the *Mo17-Rpl6a* allele. DNA sequences of PCR amplicons containing the *rpl6a::Mu-04-229 Mu* insertion region are shown. The region 3' to the *rpl6a::Mu-04-229* insertion site was amplified from total genomic DNA of the mutant with primers 1 and 5 (Fig. 1a, Table S2 in File S1). The *rpl6a::Mu-04-229* insertion region of non-mutant alleles *Rpl6a-Mo17, Rpl6a-B73* and *Rpl6a::ccB73 Mu*-on was amplified with primers 2 and 5. The PCR products were sequenced directly. The aligned sequences are shown in the sense orientation of the *rpl6a* (GRMZM2G080608-Zm00001d051422) gene model (http://www.gramene.org/, release 42, accessed 12/02/2014). (The gene model was absent in subsequent releases.) The *rpl6a::Mu-04-229 Mu* terminus, located in the exon 1 5' untranslated region, is underlined. The exon 1-intron 1 junction according to the gene model is overlined. SNPs that distinguished the *Rpl6a-Mo17* and *Rpl6a-B73* alleles, and that demonstrated *Mu* insertion into a Mo17 allele, are boxed.

```
rpl14a::Mu-04-230      CCACGCATAAGGATAGCTTGCACAGCCGTTAGAGATCAATAATAAGAAAG
Rpl14a-ccB73 Mu-on     CCACGCATAAGGATAGCTTGCACAGCCGTTAGAGATCAATAATAAGAAAG
Rpl14a-B73             CCACGCATAAGGATAGCTTGCACAGCCGTTAGAGATCAATAATAAGAAAG
Rpl14a-Mo17            CCACGCATAAGGATAGCTTGCACAGCCGTTAGAGATCAATAATAAGAAAG
                       **************************************************

rpl14a::Mu-04-230      ACGAATTCTAAATAAATAAAATGGGCTCCATATTTTAAACGATCTGGGCA
Rpl14a-ccB73 Mu-on     ACGAATTCTAAATAAATAAAATGGGCTCCATATTTTAAACGATCTGGGCA
Rpl14a-B73             ACGAATTCTAAATAAATAAAATGGGCTCCATATTTTAAACGATCTGGGCA
Rpl14a-Mo17            ACGAATTCTACATAAATAAAATGGGCTCCATATTTTAAACGAGCTGGGCA
                       ********** ******************************* *******

rpl14a::Mu-04-230      CCGCTTAACTGTAGGCCCATTACTCTATATGGGCCGGTCGACGATGTCCT
Rpl14a-ccB73 Mu-on     CCGCTTAACTGTAGGCCCATTACTCTATATGGGCCGGTCGACGATGTCCT
Rpl14a-B73             CCGCTTAACTGTAGGCCCATTACTCTATATGGGCCGGTCGACGATGTCCT
Rpl14a-Mo17            CCGCTTAACTGTAGGCCCATTACTCTATATGGGCCTGTCGACGATGTCCT
                       *********************************** **************

rpl14a::Mu-04-230      GGACAAAAACCTCCTGCAGTCGTTTTCACCTCCTCCCTCTCTCACGGGAG
Rpl14a-ccB73 Mu-on     GGACAAAAACCTCCTGCAGTCGTTTTCACCTCCTCCCTCTCTCACGGCGG
Rpl14a-B73             GGACAAAAACCTCCTGCAGTCGTTTTCACCTCCTCCCTCTCTCACGGCGG
Rpl14a-Mo17            GGACAAAAACCTCCTGCAGTCGTTTTCACCTCCTCCCTCTCTCACGGCGG
                       ***********************************************  *
```

Figure S3. The *rpl14a::Mu-04-230* allele arose by insertion into the *B73-Rpl14a* allele. DNA sequences of PCR amplicons containing the *rpl14a::Mu-04-230 Mu* insertion region are shown. Sequences 5' to the *rpl14a::Mu-04-230* insertion site were amplified from total genomic DNA of mutant plants with primers 1 and 10 (Fig. 2b, Table S2 in File S1). The *rpl14a::Mu-04-230* insertion region was amplified from non-mutant *Rpl6a-Mo17, Rpl6a-B73* and *Rpl6a-ccB73 Mu*-on alleles with primers 10 and 12. The PCR products were sequenced directly. The aligned sequences are shown in the sense orientation of the *rpl4a* gene (GRMZM2G098957-Zm00001d041322 https://ensembl.gramene.org/Zea_mays/Info/Index, accessed 8/24/2017). The underlined sequences correspond to the *Mu* terminus, located in the exon 1 5' untranslated region. Three SNPs that distinguished ccB73 and Mo17 alleles, and that demonstrated *Mu* insertion into a ccB73 allele, are boxed.

(a)

(b)

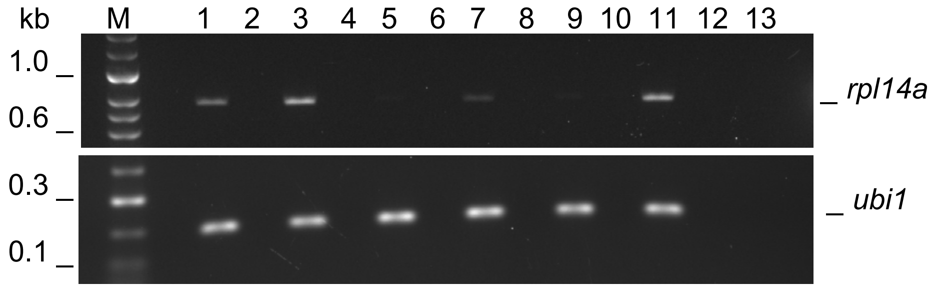

(c)

```
                            K  N  L  L  Q  S  F  S  P  P  P  S  L  T  A  A
rpl14a::Mu-1021756         ACAAAAACCTCCTGCAGTCGTTTTCACCTCCTCCCTCTCTCACGGCGGCT
rpl14a::Mu-PV03 41 D-05    ACAAAAACCTCCTGCAGTCGTTTTCACCTCCTCCCTCTCTCACGGCGGCT

                           F  L  A  T  A  A  A  A  T  T  S  S  H  L  G  K  S
rpl14a::Mu-1021756         TTTCTGGCGACGGCGGCGGCGGCGACGACGAGCAGCCACCTGGGGAAGAG
rpl14a::Mu-PV03 41 D-05    TTTCTGGCGACGGCGGCGGCGGCGACGACGAGCAGCCACCTGGGGAAGAG
                                     RPL14a
                            R  *  Q  E  M  A  A  F  L  R  S  K  C  S  P  V  G
rpl14a::Mu-1021756         CAGGTAGCAGGAAATGGCGGCGTTCCTGAGGTCAAAATGTTCACCAGTTG
rpl14a::Mu-PV03 41 D-05    CAGGTAGCAGGAAATGGCGGCGTTCCTGAGGTCAAAATGTTCACCAGTTG

                             R  T  L  M  G  S  L  G  N  S  L  F  G  A  A  N
rpl14a::Mu-1021756         GACGTACTTTGATGGGAAGCCTTGGAAATAGTTTGTTTGGGGCTGCCAAC
rpl14a::Mu-PV03 41 D-05    GACGTACTTTGATGGGAAGCCTTGGAAATAGTTTGTTTGGGGCTGCCAAC

                           S  S  V  G  A  I  T  R  P  S  H  C  D  A  I  S  Q
rpl14a::Mu-1021756         TCTTCTGTTGGGGCAATAACAAGGCCTTCTCATTGCGATGCTATCAGTCA
rpl14a::Mu-PV03 41 D-05    TCTTCTGTTGGGGCAATAACAAGGCCTTCTCATTGCGATGCTATCAGTCA

                            Q  I  R  T  F  I  Q  M  R  T  N  L  K  V  V  D  N
rpl14a::Mu-1021756         GCAAATCAGAACATTCATCCAGATGAGGACTAACCTGAAGGTGGTCGATA
rpl14a::Mu-PV03 41 D-05    GCAAATCAGAACATTCATCCAGATGAGGACTAACCTGAAGGTGGTCGATA

                             S  G  A  K  R  V  M  C  I  Q  S  L  R  G  K  K
rpl14a::Mu-1021756         ACTCCGGGGCCAAGCGGGTGATGTGCATCCAGTCCCTGAGGGGGAAGAAA
rpl14a::Mu-PV03 41 D-05    ACTCCGGGGCCAAGCGGGTGATGTGCATCCAGTCCCTGAGGGGGAAGAAA

                           G  A  R  L  G  D  M  I  I  G  S  V  K  E  A  Q  P
rpl14a::Mu-1021756         GGAGCAAGGCTCGGGGACATGATCATCGGATCCGTAAAGGAGGCCCAGCC
rpl14a::Mu-PV03 41 D-05    GGAGCAAGGCTCGGGGACATGATCATCGGATCCGTAAAGGAGGCCCAGCC

                            R  G  K  V  K  K  G  D  V  V  Y  G  V  V  V  R  A
rpl14a::Mu-1021756         TCGTGGCAAGGTCAAGAAAGGAGACGTAGTCTACGGCGTGGTCGTCCGTG
rpl14a::Mu-PV03 41 D-05    TCGTGGCAAGGTCAAGAAAGGAGACGTAGTCTACGGCGTGGTCGTCCGTG

                             A  M  K  K  G  R  S  D  G  S  E  V  Q  F  D  D
rpl14a::Mu-1021756         CCGCCATGAAGAAAGGACGCAGCGACGGCAGCGAGGTCCAGTTCGACGAC
rpl14a::Mu-PV03 41 D-05    CCGCCATGAAGAAAGGACGCAGCGACGGCAGCGAGGTCCAGTTCGACGAC

                           N  A  V  V  L  V  N  K  K  G  E  L  I  G  T  R  V
rpl14a::Mu-1021756         AACGCGGTGGTCCTCGTGAACAAGAAGGGCGAGCTGATTGGCACCCGCGT
rpl14a::Mu-PV03 41 D-05    AACGCGGTGGTCCTCGTGAACAAGAAGGGCGAGCTGATTGGCACCCGCGT

                            F  G  P  V  P  H  E  L  R  K  K  K  H  L  K  I  L
rpl14a::Mu-1021756         CTTTGGCCCCGTCCCCCACGAGCTGAGGAAGAAGAAGCACCTCAAGATCC
rpl14a::Mu-PV03 41 D-05    CTTTGGCCCCGTCCCCCACGAGCTGAGGAAGAAGAAGCACCTCAAGATCC

                             A  L  A  E  H  I  V  *
rpl14a::Mu-1021756         TGGCCCTGGCTGAACACATTGTTTGAGGTGTGTCGCATAGCCAAGTGTTT
rpl14a::Mu-PV03 41 D-05    TGGCCCTGGCTGAACACATTGTTTGAGGTGTGTCGCATAGCCAAGTGTTT
```

Figure S4. Transcripts of the *rpl4a::Mu-1021756* and *rpl4a::Mu-PV03-41-D-05* are weakly detected but correctly spliced in restored CMS-S pollen. (a) End-point reverse transcriptase PCR (RT-PCR) reaction products of pollen *rpl14a* gene transcripts fractionated by agarose gel electrophoresis are shown in panel (a) with *ubi1* gene transcript control amplifications shown in (b). Odd numbered samples contained products amplified from cDNA templates. Even numbered samples contained PCR reactions templated on cDNA synthesis controls performed in the absence of reverse transcriptase. M designates the marker lane and sample 13 contained a template-minus PCR control. Pollen genotypes used for RNA extraction were N-cytoplasm Mo17 (1 and 2), CMS-S *rpl6a::Mu-04-229* (3 and 4), CMS-S *rpl14a::Mu-04-230r39 (*5 and 6), CMS-S *rpl14a::Mu-1021756* (7 and 8), CMS-S *rpl14a::Mu-PV03 41 D-05* (9 and 10), and CMS-S Mo17 *Rf3* (11 and 12). (c) The original *rpl14a* RT-PCR products were re-amplified for DNA sequencing with primers 11 and 14 (Fig. 2a, Table S2 in File S1). cDNA sequences of *rpl4a::Mu-1021756* and *rpl4a::Mu-PV03 41 D-05* were identical and are shown aligned with their translation in the frame of the RPL14a initiation codon. The exon 1 - exon 3 junction of the transcripts is boxed and this splicing pattern was also observed in *Rpl14a-Mo17* pollen RT-PCR product sequences not shown.
